# Supplementary material for: Cultural hitchhiking and competition between patrilineal kin groups explain the post-Neolithic Y-chromosome bottleneck
Source: Nat Commun. 2018 May 25;9:2077. doi: 10.1038/s41467-018-04375-6 (PMC5970157; doi:10.1038/s41467-018-04375-6)
Supplement: Supplementary file 1 — Supplementary Information [file 41467_2018_4375_MOESM1_ESM.pdf]

# Supplementary Information

"Cultural Hitchhiking and Competition Between Patrilineal Kin Groups Explain the Post-Neolithic Y-Chromosome Bottleneck"

Zeng et al.

## Contents

**Supplementary Note 1: The Modified Lotka-Volterra Model and Proof of Claim in Results** **2**

**Supplementary Note 2: Complete Specification of Computational Grid Model** **4**

**Supplementary Note 3: Details of Grid Model Simulation and Results** **7**

**Supplementary Note 4: Sensitivity to Model Modifications** **27**

**Supplementary Note 5: Stochastic Modifications to the Modified Lotka-Volterra Model** **37**

**Supplementary Note 6: Other Computational Models** **40**

## Supplementary Note 1: The Modified Lotka-Volterra Model and Proof of Claim in Results

In Results of the main text we introduced the modified Lotka-Volterra competition model, which considers $n \geqslant 2$ populations of males competing with each other and reproducing with a common population of females. Let $Y_i$ denote the population size of male population $i$ ($i = 1, \ldots, n$), and let $X$ denote the population size of females. We assume the rate of growth of each male population is constrained by the size of all competing male populations: when an individual in population $i$ meets an individual from population $j$ (with $j \neq i$), death of an individual in population $i$ occurs at an instantaneous rate $c_{ij}$ and death of an individual in population $j$ occurs at rate $c_{ji}$. We assume population growth to be self-restraining, so that $c_{ii}$ is the instantaneous rate of death of an individual of population $i$ meeting another individual from population $i$. Further, each male population grows in proportion to the number of females $X$, with male population $i$ having a rate constant of $r_i$. Finally, we assume that $X$ grows logistically with carrying capacity and growth rate parameters $K$ and $a$ respectively. This modified Lotka-Volterra competition model is described by the following system of $n+1$ differential equations,

$$\begin{aligned}
dY_1(t) &= Y_1(t)\left(r_1X(t) - \sum_{i=1}^{n} c_{1i}Y_i(t)\right)dt \\
dY_2(t) &= Y_2(t)\left(r_2X(t) - \sum_{i=1}^{n} c_{2i}Y_i(t)\right)dt \\
&\vdots \\
dY_n(t) &= Y_n(t)\left(r_nX(t) - \sum_{i=1}^{n} c_{ni}Y_i(t)\right)dt \\
dX(t) &= aX(t)\left(1 - \frac{X(t)}{K}\right)dt
\end{aligned} \tag{S1}$$

with the following assumptions:

1. The parameters $\{r_i\}_{i=1}^n$, $\{c_{ij}\}_{1\leqslant i,j\leqslant n}$, $a$ and $K$ are positive numbers.
2. Competition between male populations is more intense than self-restraint: $c_{ij} > c_{kk}$ for all $i, j, k \in [n]$ with $i \neq j$.
3. The initial state $(Y_1(0), Y_2(0), \ldots, Y_n(0), X(0))$ lies in the section of the positive orthant $(\mathbb{R}^+_{>0})^{n+1}$ satisfying $X(0) < K$.

We claim in the Results section of the main text that when $n = 2$, one population of males almost always takes over the other. The proof is straightforward, and we include it here for completeness.

*Proof of Claim.* First, we verify that eq. 1 of the main text admits no limit cycles. Note that $X$ is an isolated autonomous system with unique solution given by

$$X(t) = \frac{KX(0)}{X(0) + (K - X(0))\exp(-at)}.$$

Hence, if $X(0) < K$, then $X(t)$ approaches the plane $X = K$. If a limit cycle exists, then it has to involve variables $Y_1$ and $Y_2$ with $X = K$. However, this is impossible as two-dimensional Lotka-Volterra systems admit no limit cycles (Hofbauer and Sigmund, 1998).

We identify the equilibria of eq. 1 of the main text. Because

$$\det\begin{pmatrix} c_{11} & c_{12} \\ c_{21} & c_{22} \end{pmatrix} < 0,$$

at most one interior equilibrium exists. For this interior equilibrium to exist in $(\mathbb{R}^+_{>0})^3$,

$$\frac{r_1}{r_2} \in \left(\frac{c_{11}}{c_{21}}, \frac{c_{12}}{c_{22}}\right) \tag{S2}$$

must hold. We assume first that eq. S2 holds and consider later the case where it does not hold.

Assuming eq. S2 holds, the only equilibria $(y_1, y_2, x)$ of the system are members of the set

$$\mathcal{E} = \left\{(0,0,0),(0,0,K),(0,\frac{r_2K}{c_{22}},K),(\frac{r_1K}{c_{11}},0,K),(\frac{r_1c_{22}-r_2c_{12}}{c_{11}c_{22}-c_{12}c_{21}}K,\frac{r_2c_{11}-r_1c_{21}}{c_{11}c_{22}-c_{12}c_{21}}K,K)\right\}.$$

To determine the local stability of each of the equilibria, we compute the Jacobian of eq. 1 of the main text,

$$J(y_1,y_2,x) = \begin{pmatrix} -2c_{11}y_1 - c_{12}y_2 + r_1x & -c_{12}y_1 & r_1y_1 \\ -c_{21}y_2 & -2c_{22}y_2 - c_{21}y_1 + r_2x & r_2y_2 \\ 0 & 0 & a - \frac{2ax}{K} \end{pmatrix}.$$

The boundary equilibria $(0, \frac{r_2K}{c_{22}}, K)$ and $(\frac{r_1K}{c_{11}}, 0, K)$ produce three negative eigenvalues, and hence are locally stable. Equilibrium $(0,0,0)$ has two zero eigenvalues and one positive eigenvalue (along the $y$ axis); it is non-hyperbolic. Equilibrium $(0,0,K)$ has two positive eigenvalues and one negative eigenvalue (along the $y$ axis) and hence is an unstable saddle. The last equilibrium, $(\frac{r_1c_{22}-r_2c_{12}}{c_{11}c_{22}-c_{12}c_{21}}K, \frac{r_2c_{11}-r_1c_{21}}{c_{11}c_{22}-c_{12}c_{21}}K, K)$, is an unstable saddle with one positive eigenvalue and two negative eigenvalues.

Thus, eq. 1 of the main text has four hyperbolic equilibria and one non-hyperbolic equilibrium, with exactly two stable equilibria, $(0, \frac{r_2K}{c_{22}}, K)$ and $(\frac{r_1K}{c_{11}}, 0, K)$. Because each unstable saddle gives rise to a locally stable submanifold, positive flows originating in $(\mathbb{R}^+_{>0})^3$ either (a) approach $(0, \frac{r_2K}{c_{22}}, K)$, or (b) approach $(\frac{r_1K}{c_{11}}, 0, K)$, or (c) approach $(0,0,K)$ if they originated along the stable submanifold of dimension 1, or (d) approach $(\frac{r_1c_{22}-r_2c_{12}}{c_{11}c_{22}-c_{12}c_{21}}K, \frac{r_2c_{11}-r_1c_{21}}{c_{11}c_{22}-c_{12}c_{21}}K, K)$ if it originated along the stable submanifold of dimension 2. The non-hyperbolicity of $(0,0,0)$ does not affect the conclusion, since all flows originating in $(\mathbb{R}^+_{>0})^3$ satisfy $\lim_{t\to+\infty} X(t) = K$. Because the Lebesgue measure of each of the stable submanifolds is 0, we conclude that flows obeying eq. 1 of the main text that originate in $(\mathbb{R}^+_{>0})^3$ converge to either $(0, \frac{r_2K}{c_{22}}, K)$ or $(\frac{r_1K}{c_{11}}, 0, K)$ almost everywhere.

If (S2) does not hold, then the interior equilibrium does not lie in $(\mathbb{R}^+_{>0})^3$. If $r_1/r_2 \leqslant c_{11}/c_{21}$, then only $(0, \frac{r_2K}{c_{22}}, K)$ is a stable sink while the other three equilibria are unstable saddles; in other words, flows originating in $(\mathbb{R}^+_{>0})^3$ lead to extinction of male population 1 almost everywhere. On the other hand, if $r_1/r_2 \geqslant c_{12}/c_{22}$, then only $(\frac{r_1K}{c_{11}}, 0, K)$ is a stable sink while the other three equilibria are unstable saddles; in other words, flows originating in $(\mathbb{R}^+_{>0})^3$ lead to extinction of male population 2 almost everywhere. This completes the proof. □

## Supplementary Note 2: Complete Specification of Computational Grid Model

The pseudocode in Supplementary Fig. 1 below completely describes the Computational Grid Model that we introduced in the Results section of the main text. The full code can be accessed online via the following link: (to be provided).

## Computational Grid Model

(A) Initialization

1. There are $|C|$ distinct cultural groups, each consisting of up to $|H|$ distinct haplogroups. Set the number of generations to $T$. (In other words, $C$ and $H$ are sets, while $T$ is a positive integer.) Set the total population size to $N^{\text{total}}$.
2. For $c \in C, h \in H, t \leqslant T$, $N_{c,h}(t)$ denotes the number of individuals belonging to cultural group $c$ and haplogroup $h$ at generation $t$.
   - If each cultural group is initially completely non-patrilineal, then $N_{c,h}(0) = N^{\text{total}}/|C|^2$ for $1 \leqslant c, h \leqslant |C|$ and $N_{c,h}(0) = 0$ otherwise. If each cultural group initially represents a patrilineal kinship group, then $N_{c,h}(0) = N^{\text{total}}/|C| > 0$ for $c = h$ and $c \leqslant |C|$ and $N_{c,h}(0)$ otherwise.
3. For $c \in C, h \in H, t \in T$, the mean number of individuals killed in class $(c, h)$ at generation $t$ follows a Poisson distribution with mean $\rho \cdot [\sum_{\{(c',h'):c' \neq c\}} N_{c',h'}(t)] \cdot \sqrt{N_{c,h}(t)}$: here, $\rho$ is a time-homogeneous scaling parameter that adjusts the mean proportion of a population killed due to competition.
4. $\mu$ is the rate of mutation per individual per generation of a majority haplogroup within each cultural group to any other haplogroup.
   - In our simulations, we assume $\mu$ is time-homogeneous. We set $\mu = 2/1000$. This is akin to the scenario in which, for every population of 1000 male individuals, every new generation produces about two new haplogroup-defining mutants.

(B) Algorithm

For $t = 0, 1, \ldots, T-1$,

1. **(Generate Random Killing Numbers)** The number of killed individuals in class $(c, h)$ is Poisson-distributed:
$$K_{c,h}(t) \sim \text{Poi}\left(\rho \cdot \left[\sum_{\{(c',h'):c' \neq c\}} N_{c',h'}(t)\right] \cdot \sqrt{N_{c,h}(t)}\right). \quad \text{(S3)}$$
2. **(Killing)** Set $x_{c,h}(t) = (N_{c,h}(t) - K_{c,h}(t)) \vee 0$.
3. **(Mutation)** For each $c \in C$,
   (i) Let $h_c$ be the haplogroup maximizing $x_{c,h}(t)$.
   (ii) Set $m_c = \mu \cdot x_{c,h_c}(t)$ and let $N^{\text{unscaled}}_{c,h_c}(t+1) = x_{c,h_c}(t) - m_c$.
   (iii) For each $h \neq h_c$, let $N^{\text{unscaled}}_{c,h}(t+1) = x_{c,h}(t) + \delta_c(h)$, where
$$\delta_c \sim \text{Multinomial}\left(m_c, \overrightarrow{\left(\frac{1}{H-1}\right)_{h \neq h_c}}\right).$$
4. **(Weeding)** For each $c \in C$,
   - If $\sum_{h \in H} N^{\text{unscaled}}_{c,h}(t+1) < 20$ (i.e., the cultural group size is too small), do:
     * Set $N^{\text{unscaled}}_{c,h}(t+1) = 0$ for all $h \in H$.
5. **(Scaling or Regeneration)** For each $c \in C, h \in H$, set
$$N_{c,h}(t+1) = N^{\text{unscaled}}_{c,h}(t+1) \cdot \frac{N^{\text{total}}}{\sum_{(c,h)} N^{\text{unscaled}}_{c,h}(t+1)}.$$
6. **(Replenishment or Group Fission)** For each $c \in C$,
   - If $\sum_{h \in H} N_{c,h}(t+1) = 0$ (i.e., the cultural group is empty), do:
     (i) Find the cultural group $c^{\max}$ that maximizes the sum $\sum_{h \in H} N_{c,h}(t+1)$.
     (ii) For each $h \in H$, move $\frac{1}{2} N_{c^{\max},h}(t+1)$ from grid cell $(c^{\max}, h)$ to grid cell $(c, h)$.

End of Algorithm.

Supplementary Figure 1: Algorithmic specification of Computational Grid Model.

We elaborate on two features of our model.

1. The mean number of a group $c$ killed per generation is proportional to the product of the total number of individuals not belonging to $c$ and the *square root* of the number of individuals belonging to $c$ (see eq. S3 of Supplementary Fig. 1). This setup differs from that of standard competition dynamics (e.g., in the Lotka-Volterra model), where the number of deaths is proportional to the number of collisions that can occur between an individual from the group and an individual from its set of competitors. We choose the square root because it results in larger cultural groups incurring fewer deaths on average, as a proportion of their size — recall that as $n$ increases, $\sqrt{n}/n = 1/\sqrt{n}$ decreases and approaches zero.

2. The haplogroup mutation rate measures the rate of "formation" of new haplogroups, which themselves arise from mutations into new haplotypes and subsequent classification. Because haplogroup classification is post-hoc and defined by groups of Y chromosomal haplotypes that share common sequence motifs at selected regions within the Y chromosome, determining the haplogroup mutation rate proves challenging.

   We suggest two approaches to approximating $\mu$ that rely on Y chromosomal data and the Y phylogeny. The first approach — the one we took to obtain the approximation $\mu = 0.002$ — starts with Y chromosome mutation rates. Taking the point mutation rate per year to be $\approx$ 7-9 $\times 10^{-10}$ (Helgason et al., 2015) and the number of years constituting one generation to be 25 (see footnote about number of generations on the next page), the mutation rate per nucleotide per generation, $m$, is 1.75-2.25$\times 10^{-8}$. Assuming each nucleotide along the complete Y chromosome mutates at roughly the same rate, the expected number of mutations along the Y chromosome per generation is $m\times$ length of Y chromosome[‡] = 0.9975-1.425, which is centered slightly above 1. Therefore, on average about 1-2 new haplotypes will emerge from point mutation in one generation, and in a population of 1000 males their offspring are expected to generate 1000 new haplotypes. However, the number of these new haplotypes that constitute haplogroups is significantly (at least of the order of $10^6$) smaller, as observed by counting the number of haplogroups on the Y tree, which spans more than $200,000$ years and grows in a population whose size is of the order of $10^6$. Our choice of $\mu$ reflects this observation, and we tested mutation rates $\mu$ that are of smaller orders, e.g., $10^{-4}$. The results of our tests yielded qualitatively similar graphs to the ones presented here. Our choice $\mu = 0.002$ also happens to agree with estimates of STR mutation rates on Y chromosomes obtained from father-son genetic studies (see Table 2 of Balanovsky, 2017).

   The second approach uses the Y tree, which is available at the International Society of Genetic Genealogy (ISOGG) or YFull (`www.yfull.com`). One may approximate the haplogroup mutation rate by estimating the average number of new haplogroups appearing per generation in a population of 1000 males over the entire length of the Y tree. The length of the Y tree is $\approx 200,000$ years, or $\approx 6,500$-$8,000$ generations with one generation spanning 25-30 years, and coalesces in an effective population size of the order of $10^6$. Denoting by $N_e(t)$ the effective population size at time $t$ (before present) and $\textsc{nh}(t)$ the number of new haplogroups appearing at time $t$, one evaluates the integral

$$\theta = 25 \times \int_0^{200000} \left( \frac{1000\textsc{nh}(t)}{N_e(t)} \right) dt,$$

   which computes the average number of new haplogroups arising per generation (= 25 years in formula above) in a population of 1000 males.

[‡] approximately $5.7 \times 10^7$ nucleotides

## Supplementary Note 3: Details of Grid Model Simulation and Results

We present results obtained from 18 distinct simulations, each sharing the following parametrization.

- $|C| = 100$ cultural groups ($C = \{1, 2, \ldots, 100\}$)
- $|H| = 500$ haplogroups ($H = \{1, 2, \ldots, 500\}$)
- Number of generations $T = 60$
- $\mu = 0.002$

We take the length of one generation, which can be interpreted as either generation time or lifespan, to be between 25 and 30 years.[‡] By setting $T = 60$ for our simulations, we are thus modeling the process over a time period of 1500-1800 years, a time length that matches the duration of the bottleneck inferred by Karmin et al. (2015). We set $|H| = 500 = 5 \times |C|$ to reflect that there are many more new haplogroups that can arise from mutation than there are cultural groups.

The 18 simulations are divided into two sets of 9 simulations, the first set simulating conditions of patrilineality and the second simulating complete non-patrilineality. To simulate conditions of patrilineality, we assume that each cultural group $c \in C$ consists of only one haplogroup at generation $T = 0$: specifically, for $c \in C$, set $N_{c,c}(0) = N^{\text{total}}/100$ and $N_{c,h}(0) = 0$ for all $h \neq c$. To simulate conditions of complete non-patrilineality, we assume that each cultural group $c \in C$ consists of equal numbers of each haplogroup up to haplogroup $|C| = 100$ at generation $T = 0$: $N_{c,1}(0) = \ldots = N_{c,100}(0) = N^{\text{total}}/100^2$.

For both the patrilineal and non-patrilineal cases, the 9 simulations specify the values of the remaining two parameters, $N^{\text{total}}$ and $\rho$. They take the following values:

1. (Total Population Size) $N^{\text{total}} \in \{10000, 20000, 30000\}$
2. (Death Rate) $\rho$, scaled to force either a $15\%, 25\%$ or $50\%$ average proportion of population decline in cultural group 1 in the beginning (i.e., $t = 0$).

As an example of how we choose the value of $\rho$, assume that $N^{\text{total}} = 10000$ and the desired proportion of population decline in the beginning is 15%. In the beginning, the total number of deaths in cultural group 1 is $D$, where (see eq. S3 in Supplementary Fig. 1)

$$D \sim \text{Poi}\left(\rho \cdot \left[\sum_{\{(c',h'):c' \neq c\}} N_{c',h'}(0)\right] \cdot \sqrt{N_{c,h}(0)}\right) = \text{Poi}(\rho \cdot 9900 \cdot 10).$$

To obtain a 15% proportion of population decline on average, we must have $\mathbb{E}[D] = (99 \times 10^3)\rho = 15$. Therefore $\rho = 15/99 \times 10^{-3} \approx 15 \times 10^{-5}$. The values of $\rho$ for the rest of the choices that we used for our simulations are specified in Table 1.

[‡] This is the range of generation times, or generation lengths, typically reported for human male populations, and it appears to be relatively stable over thousands of years. For example, pg. 229 of Barceló and Del Castillo (2016) states that the generation time for modern humans is 25 years, and a recent study relying on ancient genomes (Moorjani et al., 2016) puts the figure between 26 and 30 years for ancient populations existing 45,000 BP. A figure ranging between 20 and 35 years is often used in the evolutionary genetic literature (see, e.g., Fenner, 2005; King et al., 2006; Tremblay and Vézina, 2000). On the other hand, anthropological studies estimate the life expectancy of an individual living in Neolithic Old World population to be 26.9 at birth (Weiss and Wobst, 1973, Table 23 on pg. 49), which lies within our interval.

To summarize, our model features six parameters, four of which are shared throughout all 18 simulations ($|C|, |H|, T$ and $\mu$), and the remaining two range within a list of values to allow comparison of dynamics across different cultural group sizes (the values of $N^{\text{total}}$ itself) as well as different competition rates (the values of $\rho$, set as described above).

Supplementary Table 1 lists the 18 parametrizations that correspond to Supplementary Figs 2-19. Each figure consists of (i) a panel of graphs showing the average dynamics of the number of distinct haplogroups, haplogroup diversity, the number of distinct cultural groups, and cultural group diversity, over 100 runs sharing the specified parametrization; and (ii) graphs of the average dynamics of each of the 500 haplogroups over 100 runs, as well as the dynamics of each of the 500 haplogroups for one run. Note that because each run possibly produces a different haplogroup that dominates the distribution of existing haplogroups, we rank the haplogroups in terms of their levels of abundancy in the population after 60 generations for every run, before computing the average haplogroup dynamics across the 100 runs.

To measure diversity, we used the normalized Shannon entropy index $H^{\text{norm}}$, which is defined as follows. For a population consisting of $I$ distinct types, with type $i$ present at frequency $p_i \in (0, 1)$ for $i = 1, \ldots, I$,

$$H^{\text{norm}}(p_1, \ldots, p_I) = \frac{1}{\log I} \sum_{i=1}^{I} p_i \log \left( \frac{1}{p_i} \right).$$

Note that $H^{\text{norm}}$ ranges in $[0, 1]$ and increases as the vector of frequencies $(p_i)_{i=1}^{I}$ becomes increasingly homogeneous, with $H^{\text{norm}} = 1$ if and only if $p_1 = \ldots = p_I = 1/I$ and $H^{\text{norm}} = 0$ if and only if, for any $i = 1, \ldots, I$, $p_i = 1$ and $p_j = 0$ for each $j \neq i$.

Supplementary Table 1: List of pairs of parameters $(N^{\text{total}}, \rho)$ corresponding to choices of total population size and $\rho$ parameter. The $\rho$ parameter is calculated based on the average proportion of deaths (15%, 25% or 50%) in the first cultural group in the first generation, and is fixed after the first generation. The initial configuration (patrilineal or non-patrilineal) gives a total of 18 distinct simulations, whose figure numbers are listed in the table.

| Non-patrilineal | | | |
|---|---|---|---|
| proportion\\$N^{\text{total}}$ | 10000 | 20000 | 30000 |
| 15% | $\rho = 15 \times 10^{-5}$ (Supp. Fig. 2) | $\rho = 11 \times 10^{-5}$ (Supp. Fig. 4) | $\rho = 9 \times 10^{-5}$ (Supp. Fig. 6) |
| 25% | $\rho = 25 \times 10^{-5}$ (Supp. Fig. 8) | $\rho = 18 \times 10^{-5}$ (Supp. Fig. 10) | $\rho = 15 \times 10^{-5}$ (Supp. Fig. 12) |
| 50% | $\rho = 51 \times 10^{-5}$ (Supp. Fig. 14) | $\rho = 36 \times 10^{-5}$ (Supp. Fig. 16) | $\rho = 29 \times 10^{-5}$ (Supp. Fig. 18) |

| Patrilineal | | | |
|---|---|---|---|
| proportion\\$N^{\text{total}}$ | 10000 | 20000 | 30000 |
| 15% | $\rho = 15 \times 10^{-5}$ (Supp. Fig. 3) | $\rho = 11 \times 10^{-5}$ (Supp. Fig. 5) | $\rho = 9 \times 10^{-5}$ (Supp. Fig. 7) |
| 25% | $\rho = 25 \times 10^{-5}$ (Supp. Fig. 9) | $\rho = 18 \times 10^{-5}$ (Supp. Fig. 11) | $\rho = 15 \times 10^{-5}$ (Supp. Fig. 13) |
| 50% | $\rho = 51 \times 10^{-5}$ (Supp. Fig. 15) | $\rho = 36 \times 10^{-5}$ (Supp. Fig. 17) | $\rho = 29 \times 10^{-5}$ (Supp. Fig. 19) |

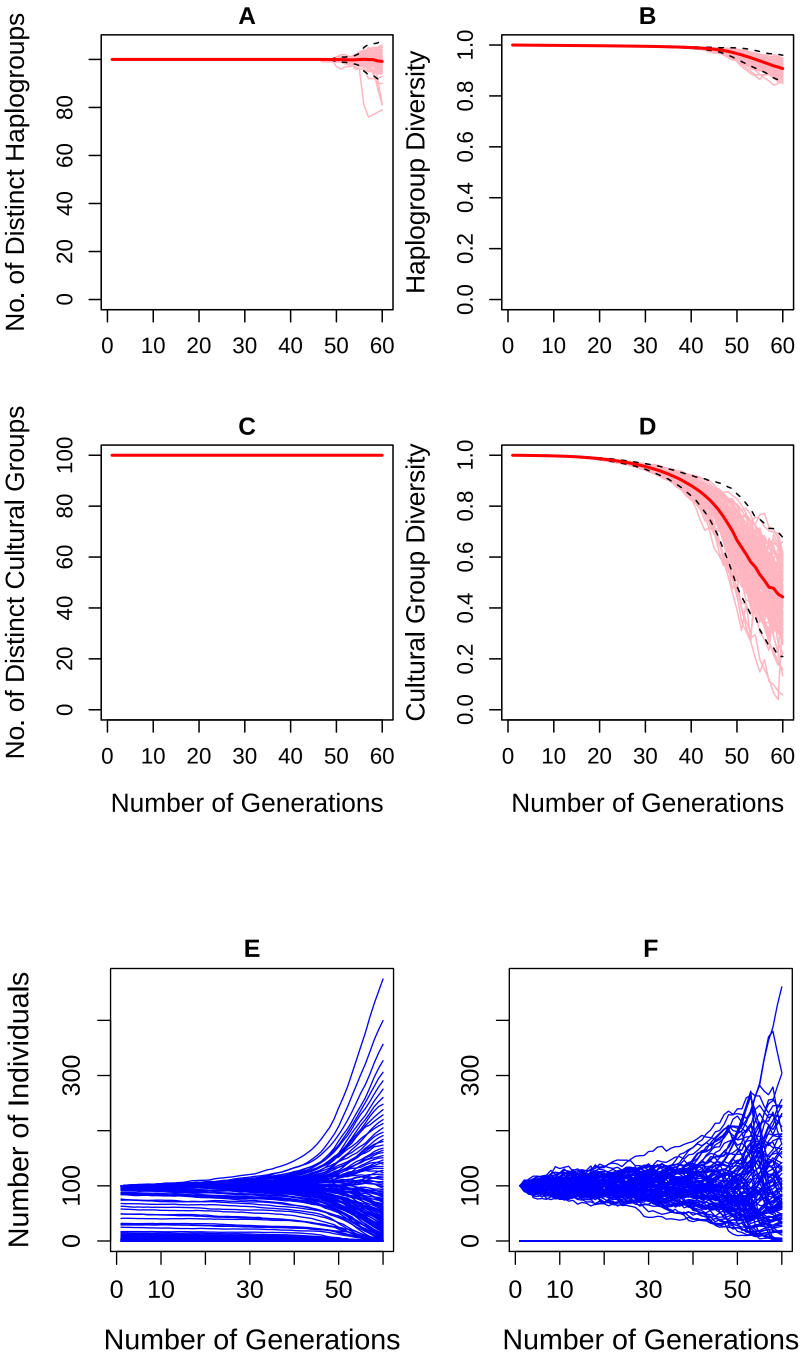


Supplementary Figure 2: Simulation assuming initial complete non-patrilineality (NPT), $N^{\text{total}} = 10000$, and a choice of $\rho$ giving rise to a 15% average population decline rate for the first cultural group initially. Because $|C| = 100$, each cultural group has size 100 and is made up of 1 member belonging to each haplogroup (1 to 100) initially. (A) Number of distinct haplogroups. (B) Haplogroup diversity. (C) Number of distinct cultural groups. (D) Cultural group diversity. (E) Average number of individuals in each haplogroup over 100 runs. (F) Number of individuals in each haplogroup for one run. Graphs (A)-(D) consist of 100 pink trajectories representing 100 distinct runs, one thick solid red trajectory representing the mean trajectory obtained by averaging across the 100 pink trajectories, and black dotted lines representing confidence intervals covering two standard deviations from the mean trajectory.

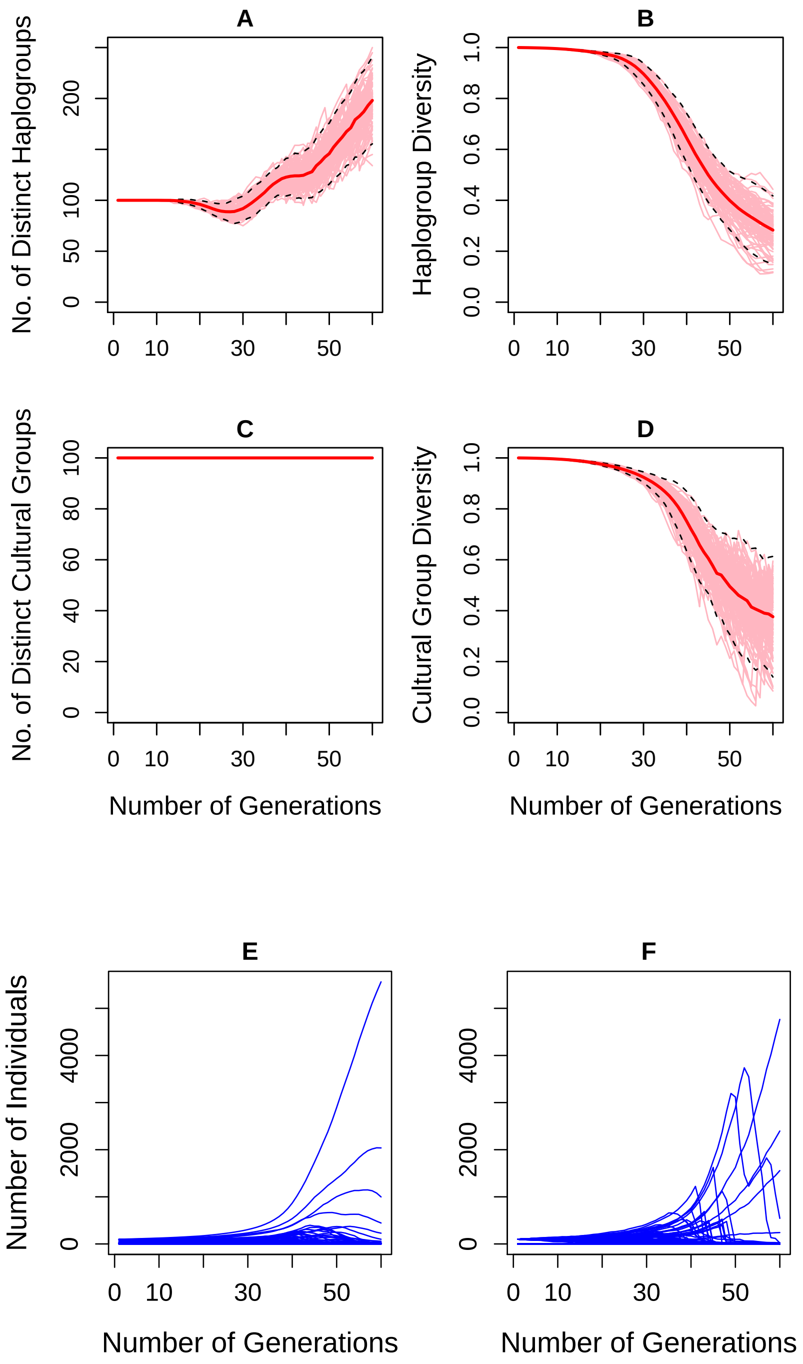


Supplementary Figure 3: Simulation assuming initial patrilineal descent groups (PT), $N^{\text{total}} = 10000$, and a choice of $\rho$ giving rise to a 15% average population decline rate for the first cultural group initially. Because $|C| = 100$, there are initially 100 patrilineal descent groups each with size 100. (A) Number of distinct haplogroups. (B) Haplogroup diversity. (C) Number of distinct cultural groups. (D) Cultural group diversity. (E) Average number of individuals in each haplogroup over 100 runs. (F) Number of individuals in each haplogroup for one run.

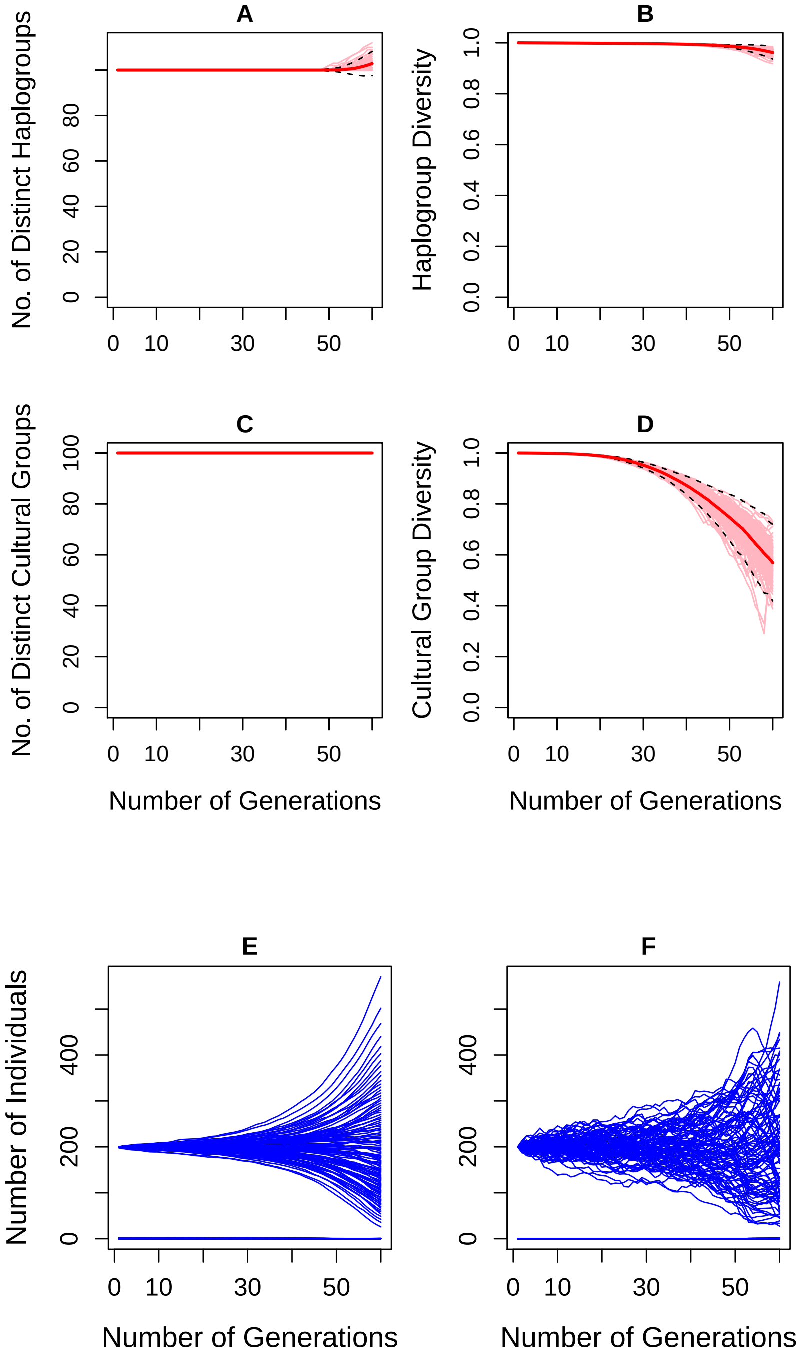


Supplementary Figure 4: Simulation assuming initial complete non-patrilineality (NPT), $N^{\text{total}} = 20000$, and a choice of $\rho$ giving rise to a 15% average population decline rate for the first cultural group initially. Because $|C| = 100$, each cultural group initially has size 200 and is made up of 2 members belonging to each haplogroup (1 to 100). (A) Number of distinct haplogroups. (B) Haplogroup diversity. (C) Number of distinct cultural groups. (D) Cultural group diversity. (E) Average number of individuals in each haplogroup over 100 runs. (F) Number of individuals in each haplogroup for one run.

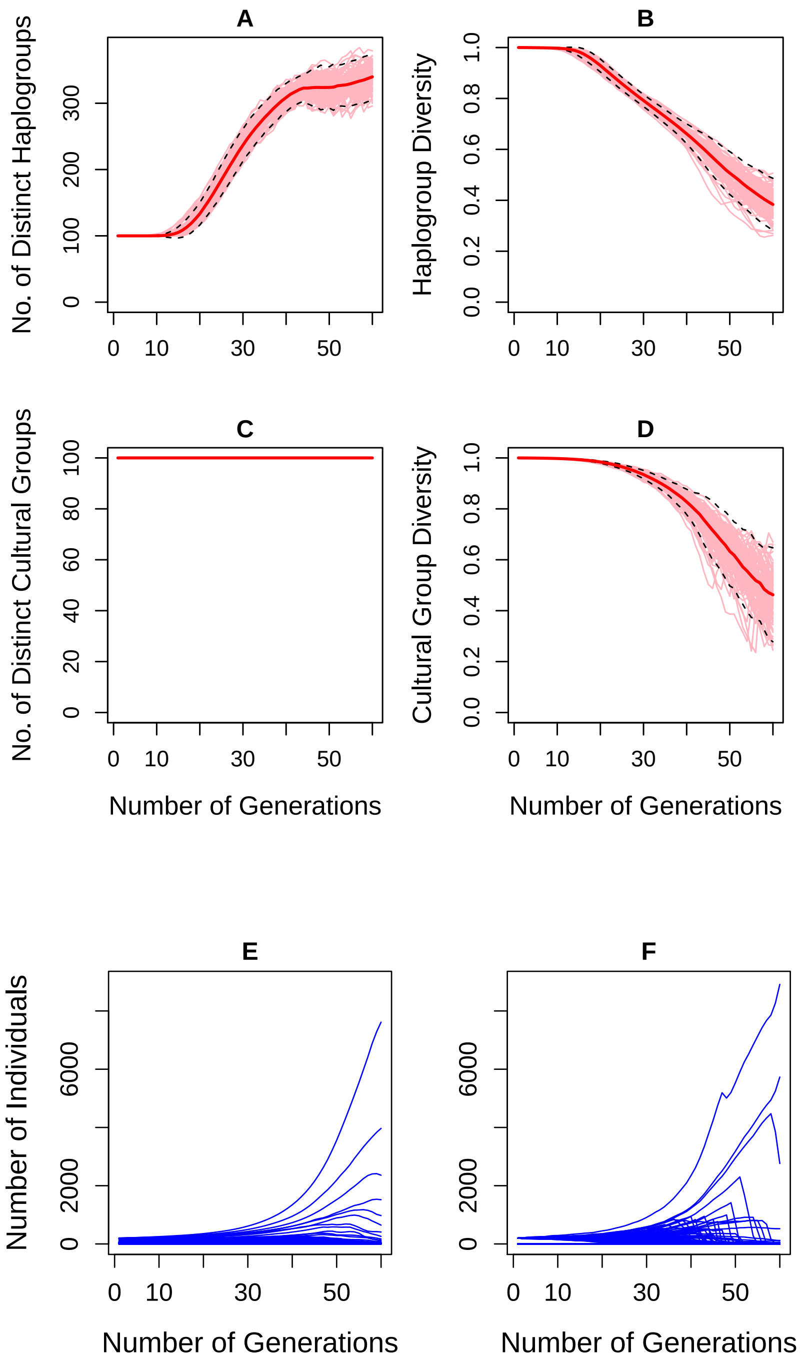


Supplementary Figure 5: Simulation assuming initial patrilineal descent groups (PT), $N^{\text{total}} = 20000$, and a choice of $\rho$ giving rise to a 15% average population decline rate for the first cultural group initially. Because $|C| = 100$, there are initially 100 patrilineal descent groups each with size 200. (A) Number of distinct haplogroups. (B) Haplogroup diversity. (C) Number of distinct cultural groups. (D) Cultural group diversity. (E) Average number of individuals in each haplogroup over 100 runs. (F) Number of individuals in each haplogroup for one run.

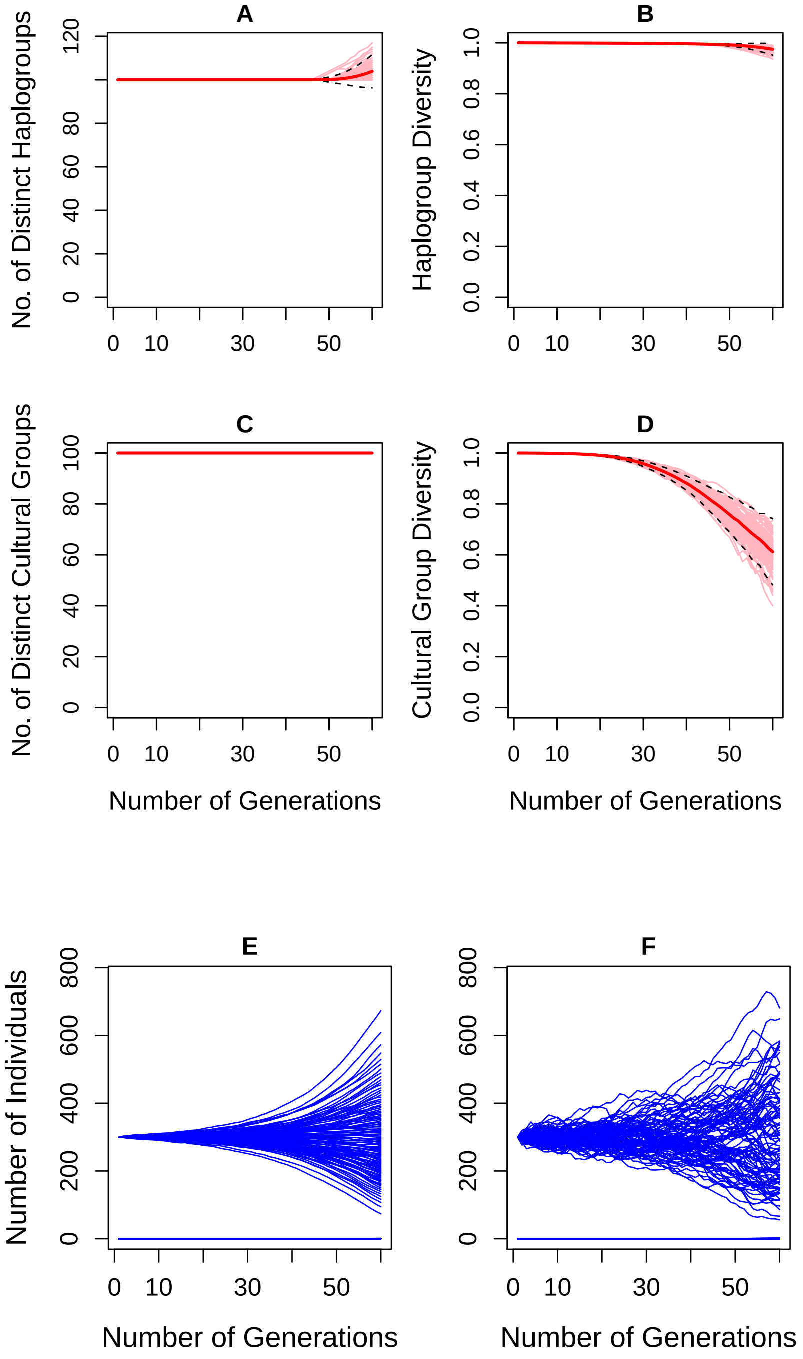


Supplementary Figure 6: Simulation assuming initial complete non-patrilineality (NPT), $N^{\text{total}} = 30000$, and a choice of $\rho$ giving rise to a 15% average population decline rate for the first cultural group initially. Because $|C| = 100$, each cultural group initially has size 300 and is made up of 3 members belonging to each haplogroup (1 to 100). (A) Number of distinct haplogroups. (B) Haplogroup diversity. (C) Number of distinct cultural groups. (D) Cultural group diversity. (E) Average number of individuals in each haplogroup over 100 runs. (F) Number of individuals in each haplogroup for one run.

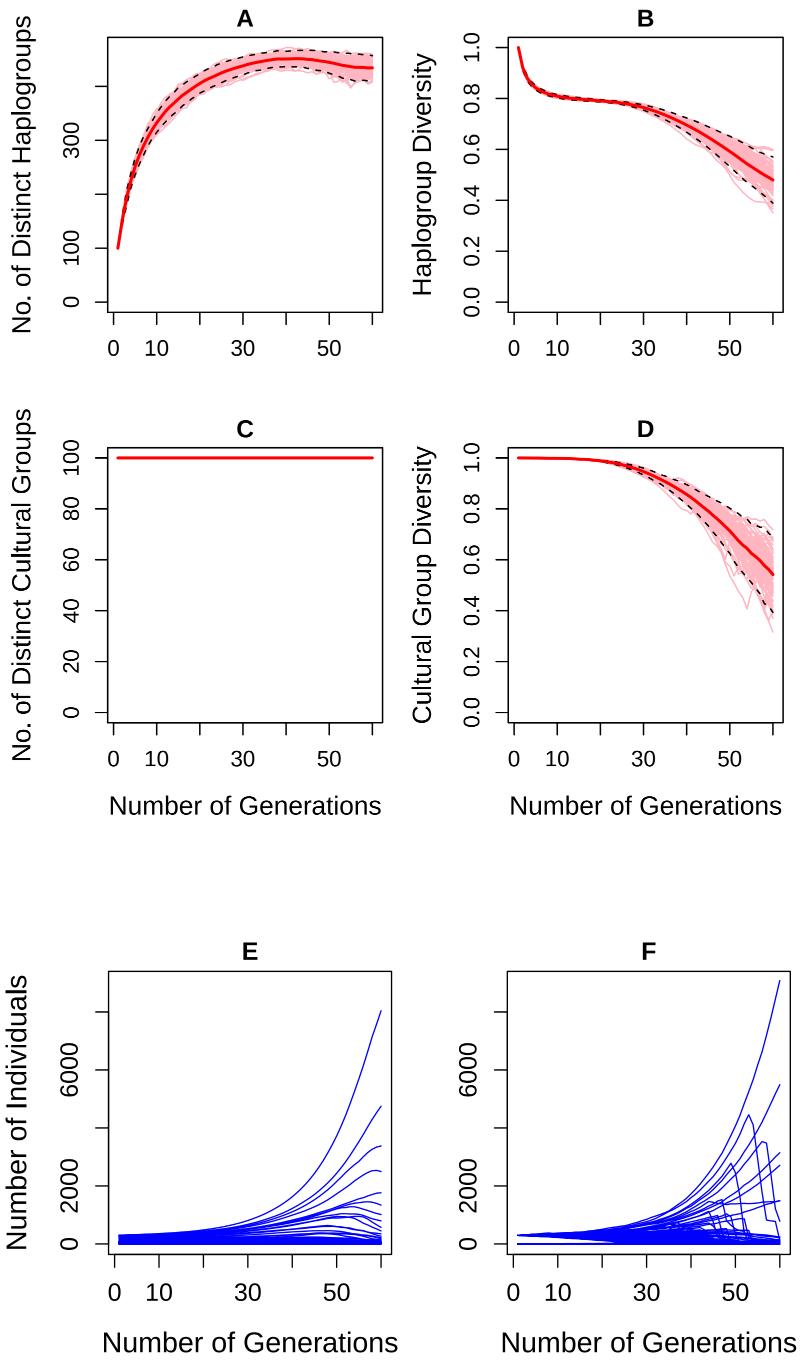


Supplementary Figure 7: Simulation assuming initial patrilineal descent groups (PT), $N^{\text{total}} = 30000$, and a choice of $\rho$ giving rise to a 15% average population decline rate for the first cultural group initially. Because $|C| = 100$, there are initially 100 patrilineal descent groups each with size 300. (A) Number of distinct haplogroups. (B) Haplogroup diversity. (C) Number of distinct cultural groups. (D) Cultural group diversity. (E) Average number of individuals in each haplogroup over 100 runs. (F) Number of individuals in each haplogroup for one run.

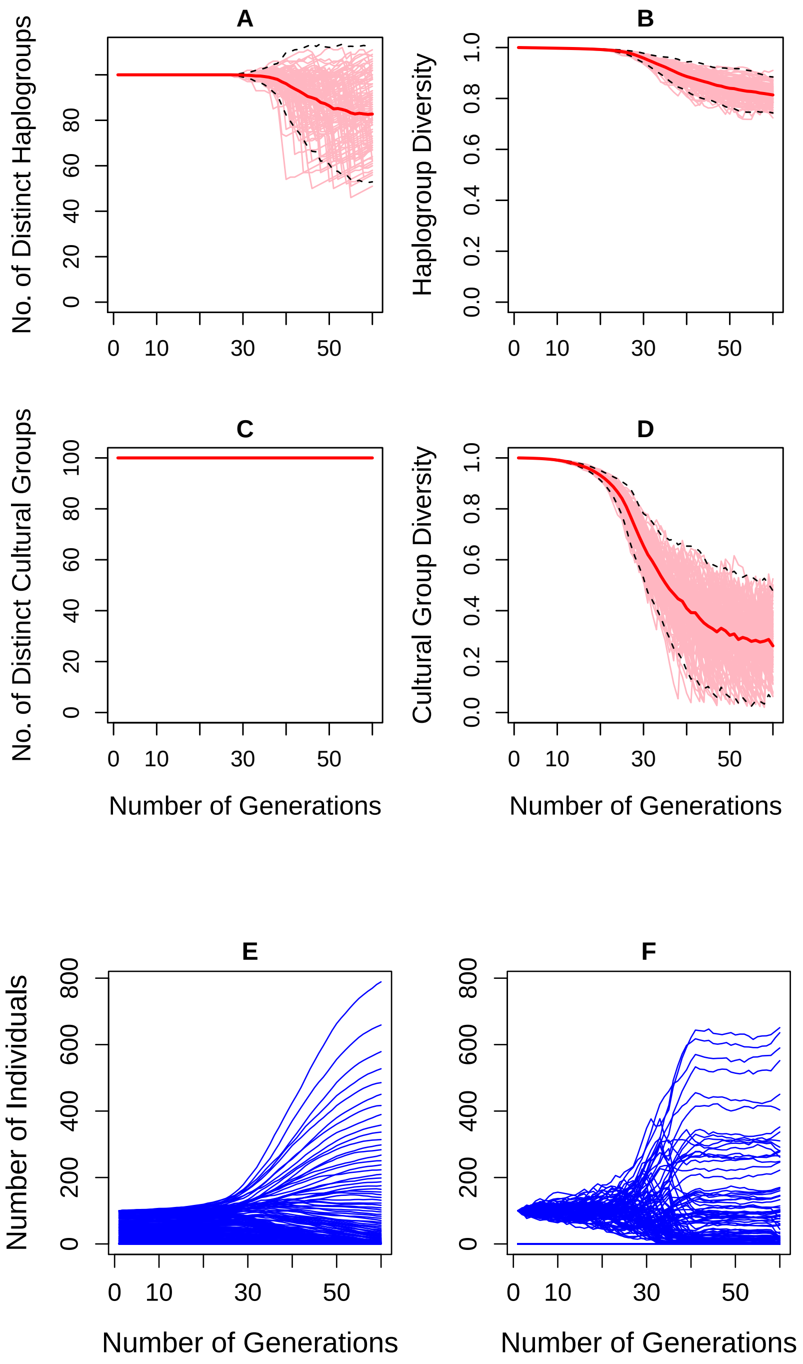


Supplementary Figure 8: Simulation assuming initial complete non-patrilineality (NPT), $N^{\text{total}} = 10000$, and a choice of $\rho$ giving rise to a 25% average population decline rate for the first cultural group initially. Because $|C| = 100$, each cultural group initially has size 100 and is made up of 1 member belonging to each haplogroup (1 to 100). (A) Number of distinct haplogroups. (B) Haplogroup diversity. (C) Number of distinct cultural groups. (D) Cultural group diversity. (E) Average number of individuals in each haplogroup over 100 runs. (F) Number of individuals in each haplogroup for one run.

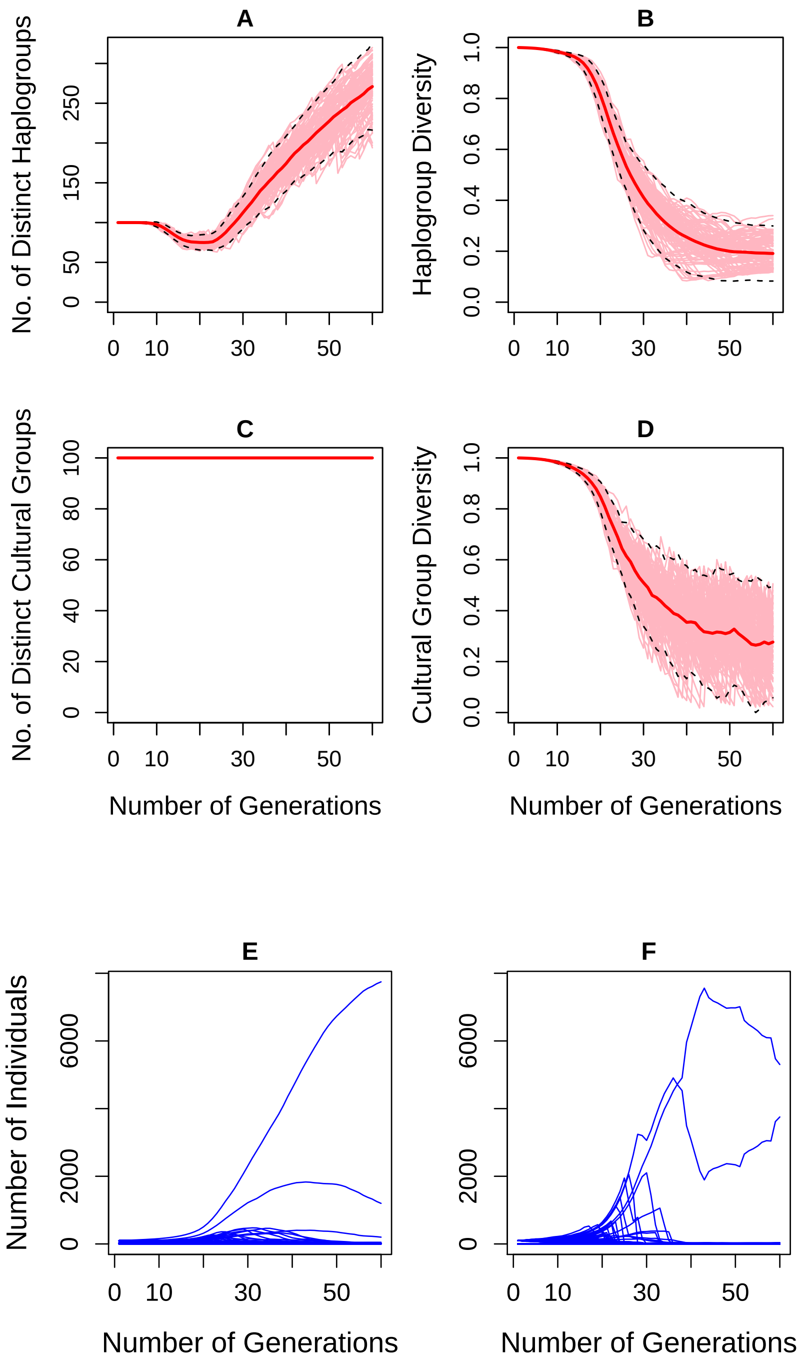


Supplementary Figure 9: Simulation assuming initial patrilineal descent groups (PT), $N^{\text{total}} = 10000$, and a choice of $\rho$ giving rise to a 25% average population decline rate for the first cultural group initially. Because $|C| = 100$, there are initially 100 patrilineal descent groups each with size 100. (A) Number of distinct haplogroups. (B) Haplogroup diversity. (C) Number of distinct cultural groups. (D) Cultural group diversity. (E) Average number of individuals in each haplogroup over 100 runs. (F) Number of individuals in each haplogroup for one run.

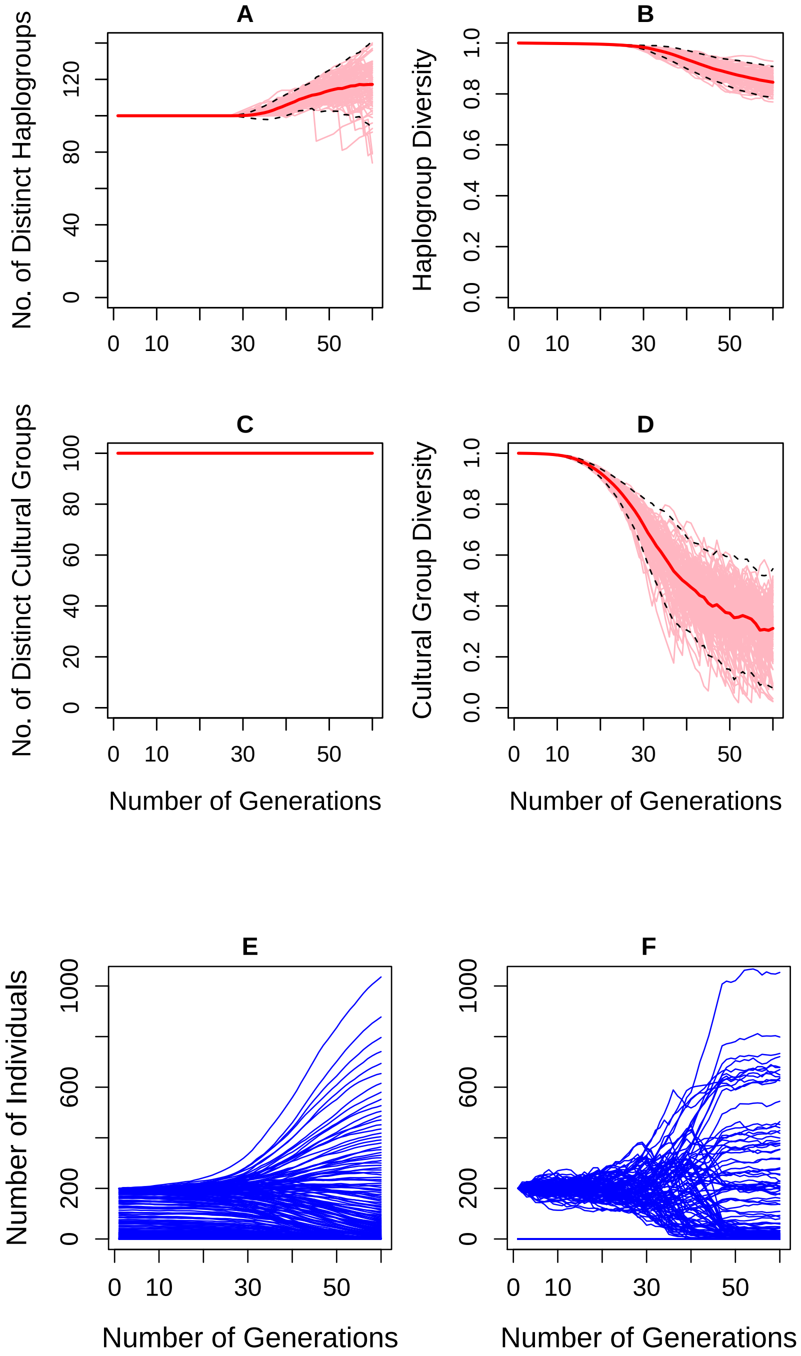


Supplementary Figure 10: Simulation assuming initial complete non-patrilineality (NPT), $N^{\text{total}} = 20000$, and a choice of $\rho$ giving rise to a 25% average population decline rate for the first cultural group initially. Because $|C| = 100$, each cultural group initially has size 200 and is made up of 2 members belonging to each haplogroup (1 to 100). (A) Number of distinct haplogroups. (B) Haplogroup diversity. (C) Number of distinct cultural groups. (D) Cultural group diversity. (E) Average number of individuals in each haplogroup over 100 runs. (F) Number of individuals in each haplogroup for one run.

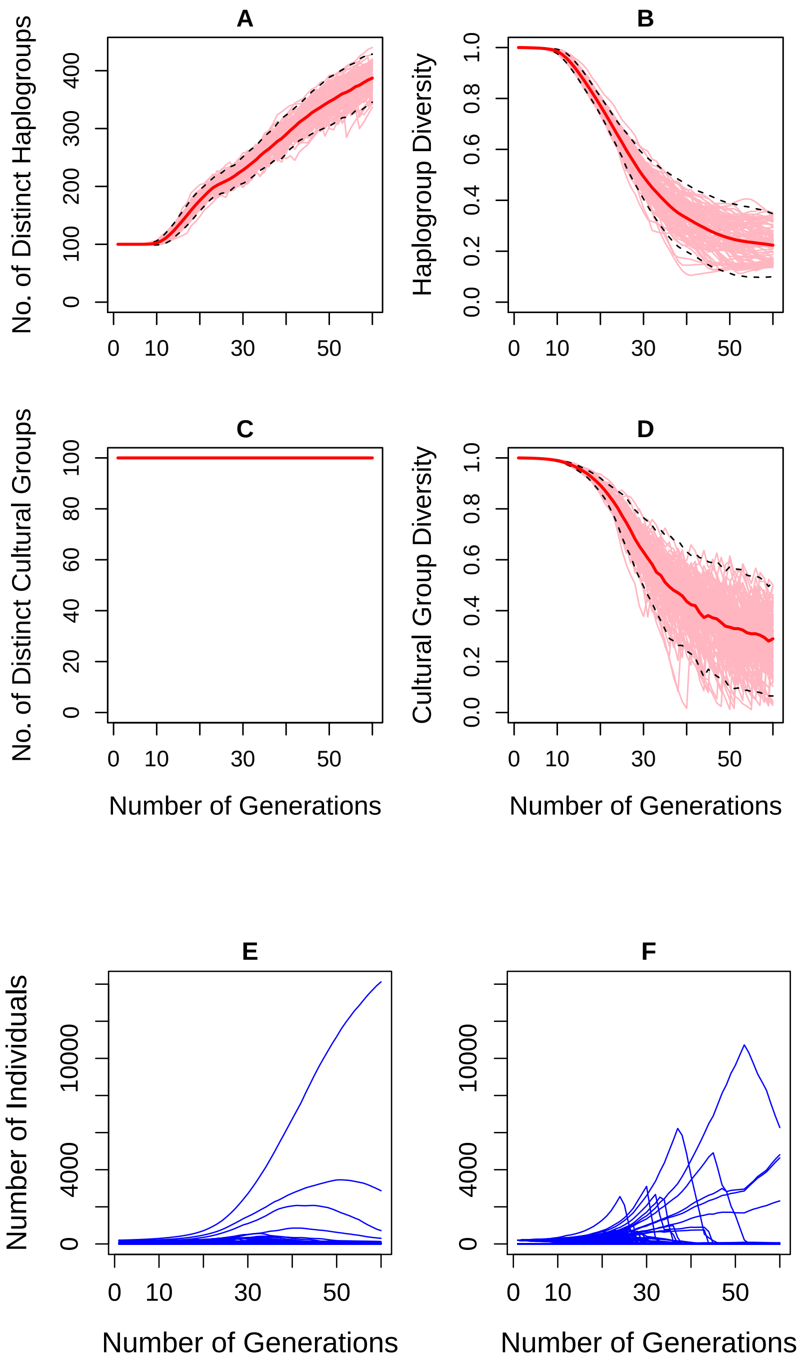


Supplementary Figure 11: Simulation assuming initial patrilineal descent groups (PT), $N^{\text{total}} = 20000$, and a choice of $\rho$ giving rise to a 25% average population decline rate for the first cultural group initially. Because $|C| = 100$, there are initially 100 patrilineal descent groups each with size 200. (A) Number of distinct haplogroups. (B) Haplogroup diversity. (C) Number of distinct cultural groups. (D) Cultural group diversity. (E) Average number of individuals in each haplogroup over 100 runs. (F) Number of individuals in each haplogroup for one run.

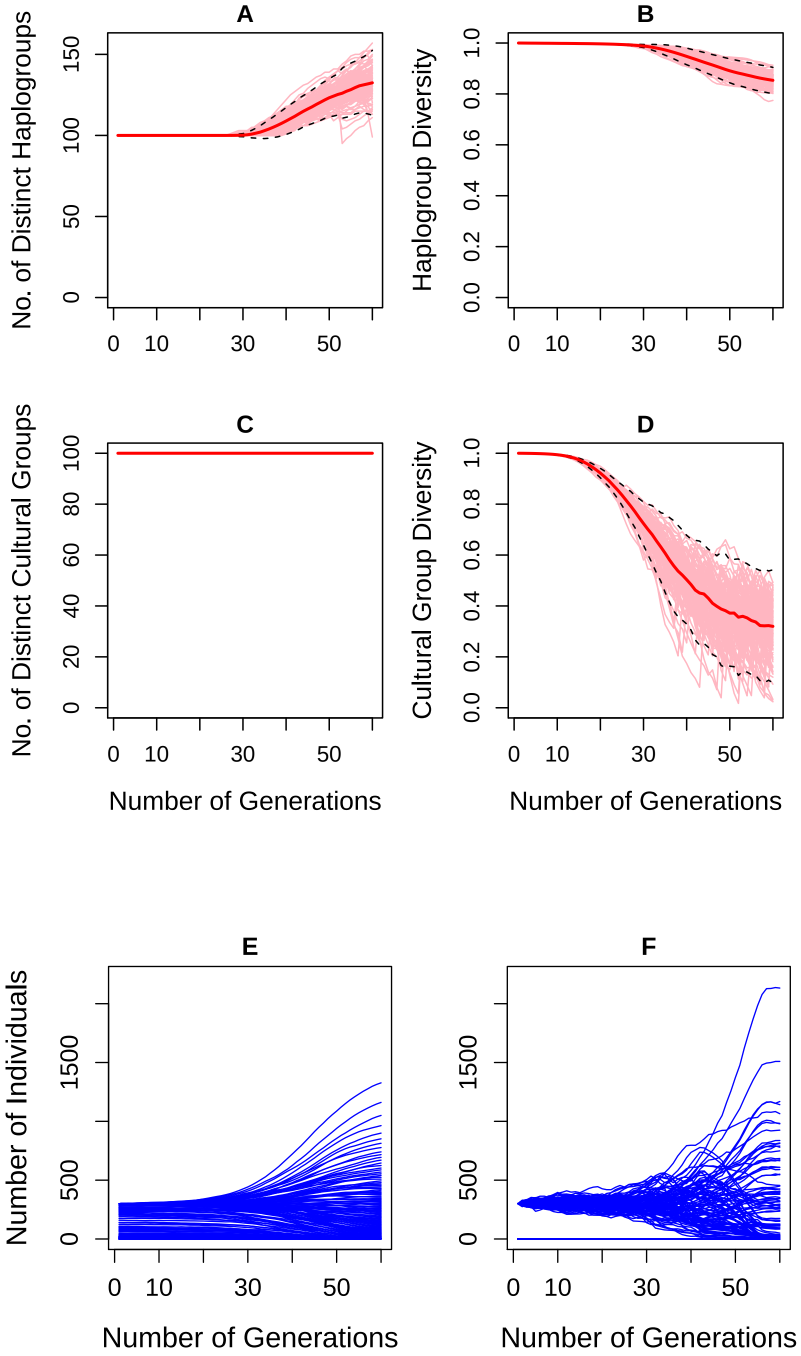


Supplementary Figure 12: Simulation assuming initial complete non-patrilineality (NPT), $N^{\text{total}} = 30000$, and a choice of $\rho$ giving rise to a 25% average population decline rate for the first cultural group initially. Because $|C| = 100$, each cultural group initially has size 300 and is made up of 3 members belonging to each haplogroup (1 to 100). (A) Number of distinct haplogroups. (B) Haplogroup diversity. (C) Number of distinct cultural groups. (D) Cultural group diversity. (E) Average number of individuals in each haplogroup over 100 runs. (F) Number of individuals in each haplogroup for one run.

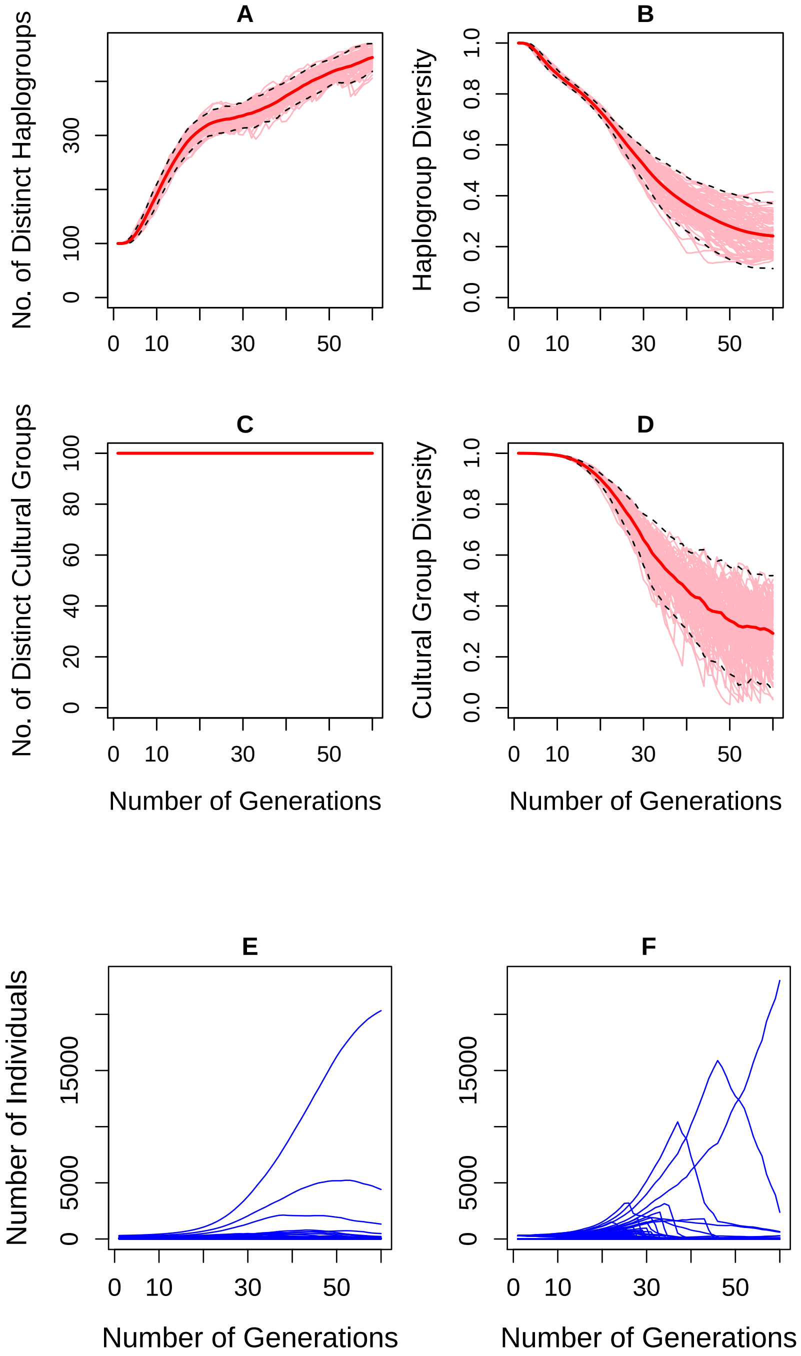


Supplementary Figure 13: Simulation assuming initial patrilineal descent groups (PT), $N^{\text{total}} = 30000$, and a choice of $\rho$ giving rise to a 25% average population decline rate for the first cultural group initially. Because $|C| = 100$, there are initially 100 patrilineal descent groups each with size 300. (A) Number of distinct haplogroups. (B) Haplogroup diversity. (C) Number of distinct cultural groups. (D) Cultural group diversity. (E) Average number of individuals in each haplogroup over 100 runs. (F) Number of individuals in each haplogroup for one run.

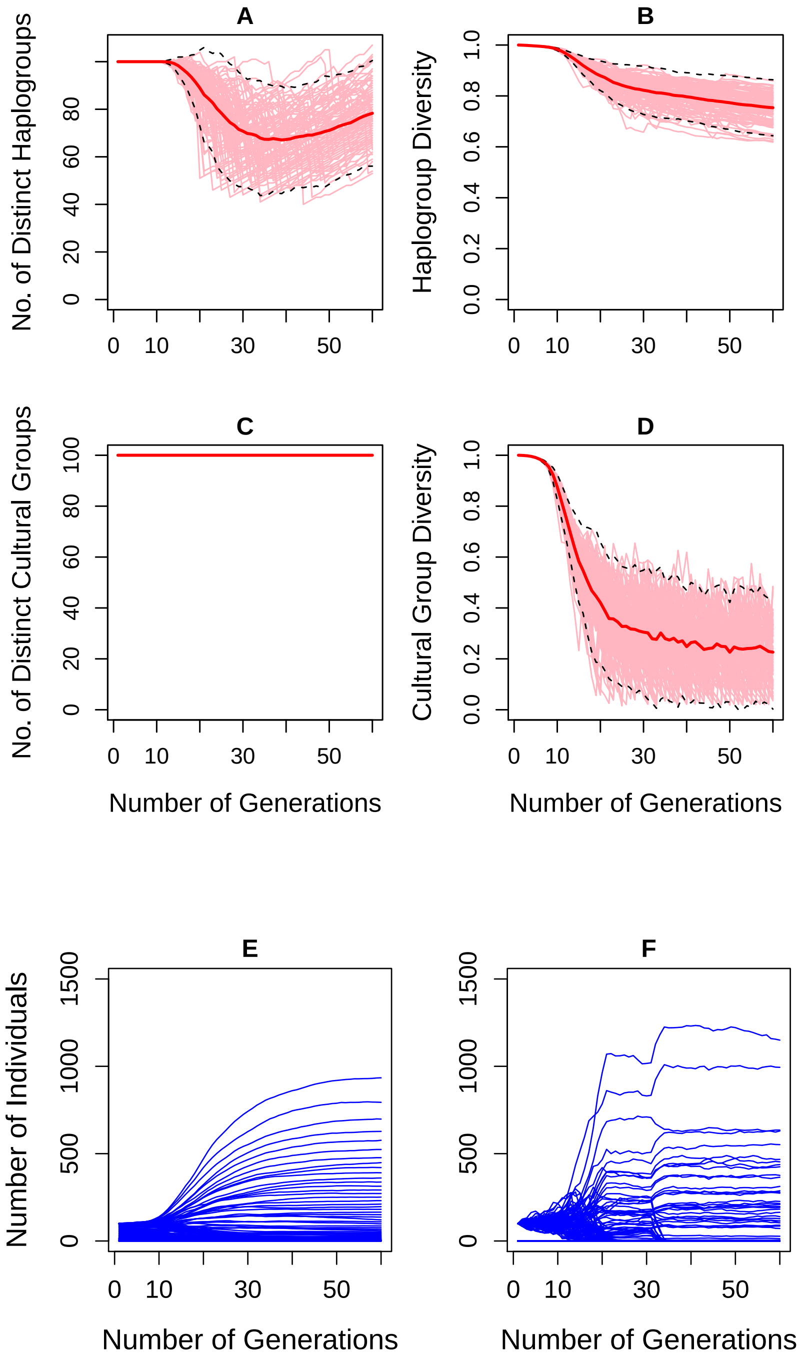


Supplementary Figure 14: Simulation assuming initial complete non-patrilineality (NPT), $N^{\text{total}} = 10000$, and a choice of $\rho$ giving rise to a 50% average population decline rate for the first cultural group initially. Because $|C| = 100$, each cultural group initially has size 100 and is made up of 1 member belonging to each haplogroup (1 to 100). (A) Number of distinct haplogroups. (B) Haplogroup diversity. (C) Number of distinct cultural groups. (D) Cultural group diversity. (E) Average number of individuals in each haplogroup over 100 runs. (F) Number of individuals in each haplogroup for one run.

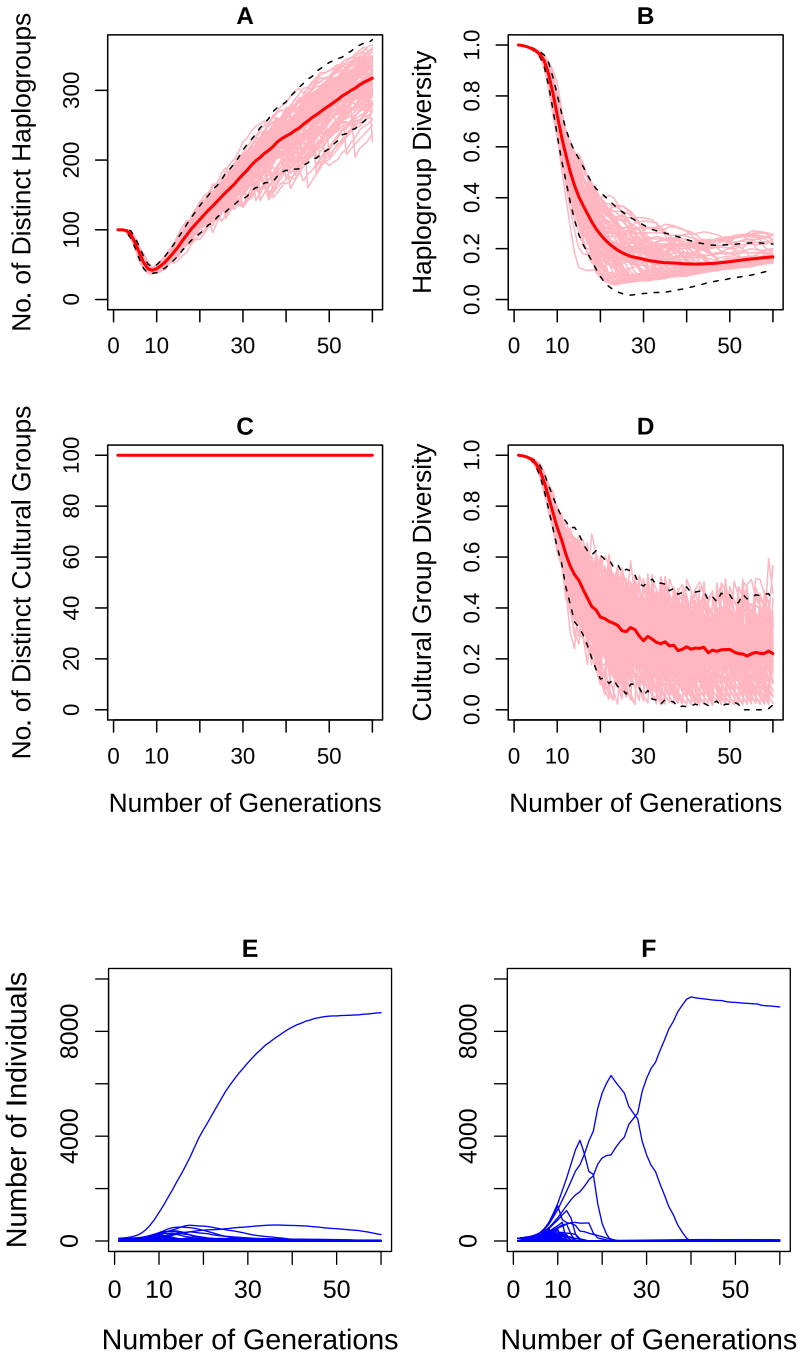


Supplementary Figure 15: Simulation assuming initial patrilineal descent groups (PT), $N^{\text{total}} = 10000$, and a choice of $\rho$ giving rise to a 50% average population decline rate for the first cultural group initially. Because $|C| = 100$, there are initially 100 patrilineal descent groups each with size 100. (A) Number of distinct haplogroups. (B) Haplogroup diversity. (C) Number of distinct cultural groups. (D) Cultural group diversity. (E) Average number of individuals in each haplogroup over 100 runs. (F) Number of individuals in each haplogroup for one run.

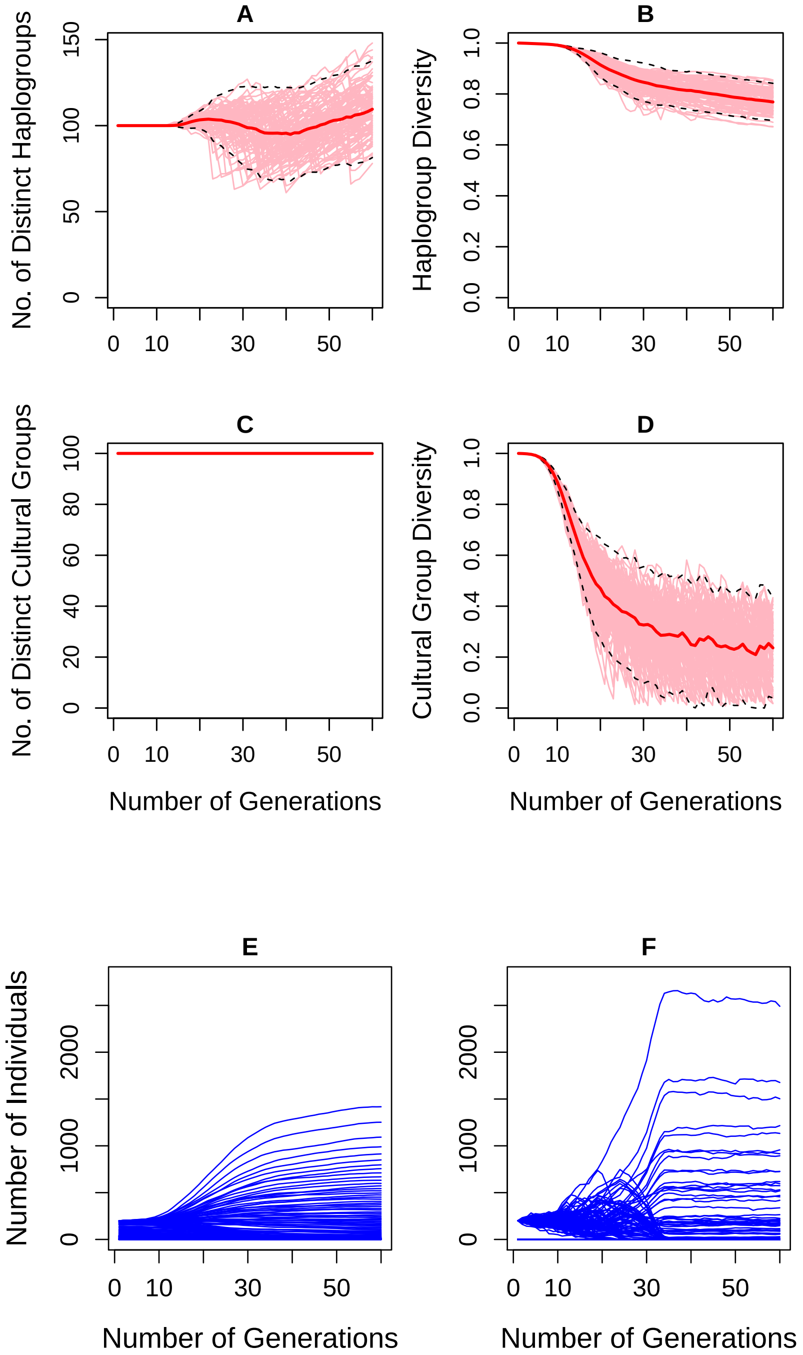


Supplementary Figure 16: Simulation assuming initial complete non-patrilineality (NPT), $N^{\text{total}} = 20000$, and a choice of $\rho$ giving rise to a 50% average population decline rate for the first cultural group initially. Because $|C| = 100$, each cultural group initially has size 200 and is made up of 2 members belonging to each haplogroup (1 to 100). (A) Number of distinct haplogroups. (B) Haplogroup diversity. (C) Number of distinct cultural groups. (D) Cultural group diversity. (E) Average number of individuals in each haplogroup over 100 runs. (F) Number of individuals in each haplogroup for one run.

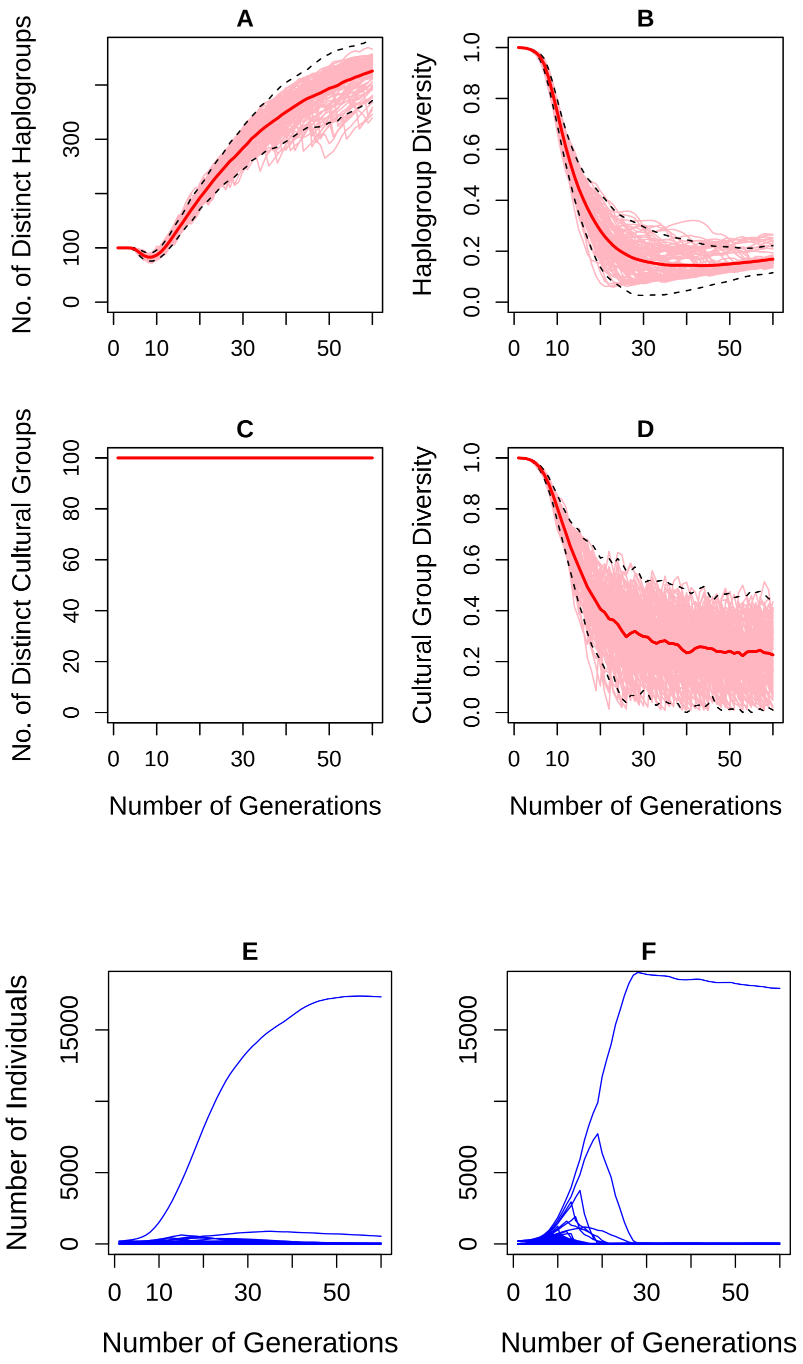


Supplementary Figure 17: Simulation assuming initial patrilineal descent groups (PT) at the beginning, $N^{\text{total}} = 20000$, and a choice of $\rho$ giving rise to a 50% average population decline rate for the first cultural group initially. Because $|C| = 100$, there are initially 100 patrilineal descent groups each with size 200. (A) Number of distinct haplogroups. (B) Haplogroup diversity. (C) Number of distinct cultural groups. (D) Cultural group diversity. (E) Average number of individuals in each haplogroup over 100 runs. (F) Number of individuals in each haplogroup for one run.

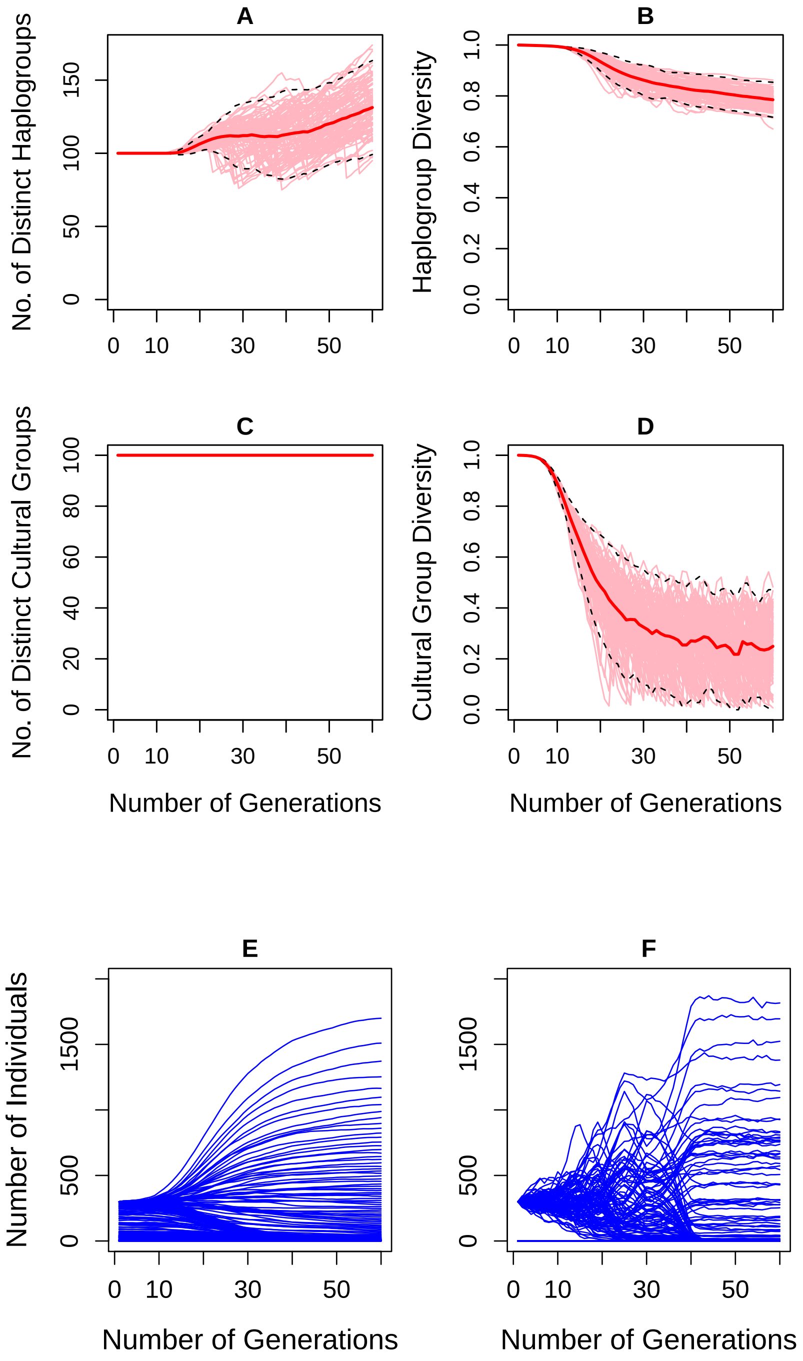


Supplementary Figure 18: Simulation assuming initial complete non-patrilineality (NPT), $N^{\text{total}} = 30000$, and a choice of $\rho$ giving rise to a 50% average population decline rate for the first cultural group initially. Because $|C| = 100$, each cultural group initially has size 300 and is made up of 3 members belonging to each haplogroup (1 to 100). (A) Number of distinct haplogroups. (B) Haplogroup diversity. (C) Number of distinct cultural groups. (D) Cultural group diversity. (E) Average number of individuals in each haplogroup over 100 runs. (F) Number of individuals in each haplogroup for one run.

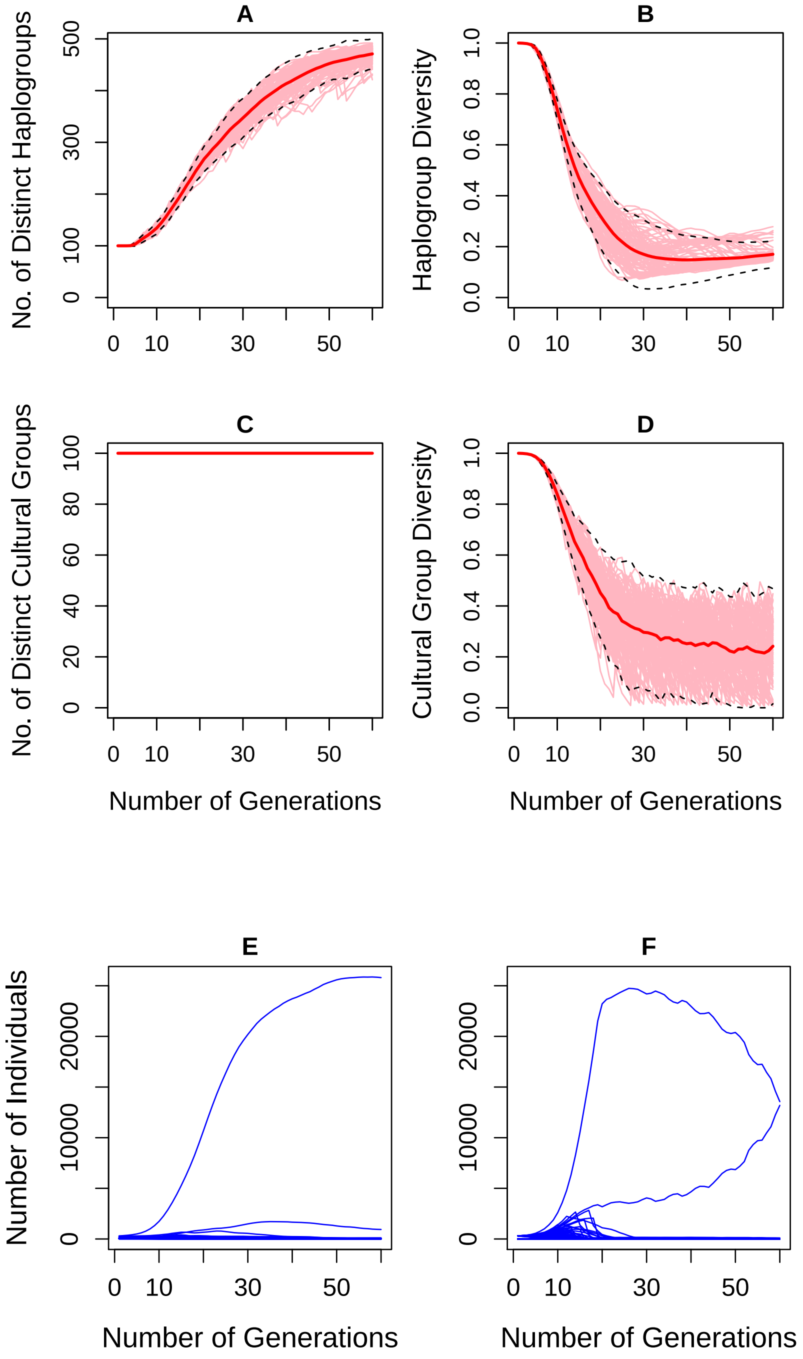


Supplementary Figure 19: Simulation assuming initial patrilineal descent groups (PT), $N^{\text{total}} = 30000$, and a choice of $\rho$ giving rise to a 50% average population decline rate for the first cultural group initially. Because $|C| = 100$, there are initially 100 patrilineal descent groups each with size 300. (A) Number of distinct haplogroups. (B) Haplogroup diversity. (C) Number of distinct cultural groups. (D) Cultural group diversity. (E) Average number of individuals in each haplogroup over 100 runs. (F) Number of individuals in each haplogroup for one run.

## Supplementary Note 4: Sensitivity to Model Modifications

Our computational model encompasses our hypothesis about the sociocultural process that resulted in the bottleneck. Treating it as a "null model," we describe how our model can be modified to reflect different anthropological and sociological conditions. These include (1) changing the ratio of the number of cultural groups to the number of haplogroups ($|C| : |H|$ ratio), (2) incorporating selection among cultural groups, and (3) including fusion of cultural groups. In each modification parameters are changed. We refer to these model modification parameters as "hyperparameters" to distinguish them from the parameters described in Supplementary Note 3. We elaborate on these hyperparameter changes below.

1. **(Changing the $|C| : |H|$ Ratio)** In our null hypothesis, we fixed $|C| : |H| = 1 : 5$. This ratio can be modified; as an example we present simulations in which $|C| = 100$ but $|H| = 2500$, shifting the ratio downward, which more closely mirrors an infinite alleles model.

2. **(Incorporating Selection)** Our null hypothesis assumes no fitness differences among cultural groups. It is very plausible that in addition to competition in which size alone confers survival advantage — so-called selection for large groups (see Henrich, 2004) — differences in cultural innovations or toolkits act as a secondary mechanism of selection among these groups. To incorporate such selection effects, we modify the method by which the numbers killed are generated, i.e., eq. (3) of Supplementary Fig. 1. Instead of

$$K_{c,h}(t) \sim \text{Poi}\left(\rho \cdot \left[\sum_{\{(c',h'):c'\neq c\}} N_{c',h'}(t)\right] \cdot \sqrt{N_{c,h}(t)}\right),$$

we set

$$K_{c,h}(t) \sim \text{Poi}\left(\alpha_c \cdot \rho \cdot \left[\sum_{\{(c',h'):c'\neq c\}} N_{c',h'}(t)\right] \cdot \sqrt{N_{c,h}(t)}\right),$$

where $\{\alpha_c : c \in C\}$ are parameters describing the fitness differentials among cultural groups. As an example, we assume that the cultural groups are linearly ordered in terms of their relative fitnesses, with the first cultural group being the fittest and subsequent groups' fitnesses declining. Because the fittest cultural group suffers from the fewest number of deaths, $\alpha_c$ increases in $c$. We consider $\alpha_c = 1.00 + (c - 1) \times 0.01$; in other words normalizing the fitness differential so that the fittest cultural group (namely, cultural group $c = 1$) suffers the same mean proportion of deaths as it does in our null model and subsequent cultural groups suffer a greater mean proportion of deaths. Our choice of a linear function for $\alpha_c$ is for simplicity.

3. **(Including Group Fusion)** Step 4 of the Computational Grid Model (Supplementary Fig. 1) simply removes inviably small cultural groups. In reality, however, small cultural groups may be assimilated into larger ones. We can modify Step 4 to incorporate such group fusion mechanisms; as an example we use a stepping-stone approach, moving individuals in each small cultural group (i.e., whose size is $< 20$) to either the cultural group above it or below it in our grid. If this occurs to the last cultural group, we allow movement to the first cultural group; if this occurs to the first cultural group, we allow movement to the last cultural group. Note that in moving these individuals, their haplogroups do not change — in other words, we are effectively shifting a row of the grid up or down.

To demonstrate the effects of changing the three hyperparameters, we perform simulations incorporating (1) exactly one of the three hyperparameter changes; (2) two of the three hyperparameter changes; and (3) all three hyperparameter changes. We do so using the parameter setting $(N^{\text{total}}, \rho) = (20000, 18 \times 10^{-5})$, which is the case of 100 cultural groups each having size 200 and with mean proportion of deaths in the first generation approximately 25%. Note that Supplementary Figs 10 and 11 show the dynamics under this parametrization for our null hypothesis. Supplementary Table 2 lists figures corresponding to simulations performed with various combinations of hyperparameter changes. As in Supplementary Figs 2-19, we produce six graphs summarizing the dynamics of cultural group and haplogroup diversity and richness, as well as average and single run dynamics of the individual haplogroups in $H$. Additionally, for each combination of modifications, we present together results of simulations for both nonpatrilineal (NPT) and patrilineal (PT) configurations.

Supplementary Table 2: List of figures corresponding to modifications made to the null model.

| Modification | Reference Figure |
|---|---|
| Changing $\lvert C\rvert : \lvert H\rvert$ ratio | Supp. Fig. 20 |
| Incorporating selection | Supp. Fig. 21 |
| Including group fusion | Supp. Fig. 22 |
| Changing $\lvert C\rvert : \lvert H\rvert$ ratio, incorporating selection | Supp. Fig. 23 |
| Changing $\lvert C\rvert : \lvert H\rvert$ ratio, including group fusion | Supp. Fig. 24 |
| Incorporating selection, including group fusion | Supp. Fig. 25 |
| Changing $\lvert C\rvert : \lvert H\rvert$ ratio, incorporating selection, including group fusion | Supp. Fig. 26 |

We note the following with regard to changing hyperparameters. First, by changing the $|C| : |H|$ ratios (Supplementary Fig. 20), we observe no changes in the qualitative outcome of the simulations: patrilineal configurations lead to the death of most lineages and the dominance of few — typically less than four — major lineages, while non-patrilineality leads to the survival of multiple haplogroup lineages, with lineage sizes all of the same order of magnitude. Second, by incorporating selection, via a culture-related fitness differential (Supplementary Fig. 21), we find that there is a quicker spreading out of haplogroup lineages, and these lineages eventually vanish or persist in the same qualitative manner as they do in our original simulations. Moreover, in the patrilineal initial configuration case, not only is there less variation in haplogroup richness and haplogroup diversity, but also fewer haplogroup lineages persist in large numbers, either on average or in a single run. Third, including group fusion through a stepping-stone mechanism (Supplementary Fig. 22) produces no qualitative changes in simulation outcomes for both patrilineal and non-patrilineal initial configurations.

We observe little change despite applying multiple modifications at once, as exemplified by the dynamics shown in Supplementary Figs 23-26. The qualitative outcomes of simulations starting from either patrilineal or non-patrilineal configurations appear robust to the modifications described above.

These changes in the model sharpen the questions we can ask about the processes that drove the persistence of the bottleneck. For instance, the strength of selection pressure on cultural groups —

captured by the gradient of the fitness differential $\alpha_c$ and its choice (not necessarily a linear function, as assumed in our simulations) — can be studied as a covariate influencing the speed of divergence of haplogroup sizes. This helps lay the foundation for the development of a statistical framework to infer the degree of selection (or fusion) present in populations that historically experienced intergroup competition and whose present-day genome samples are available.

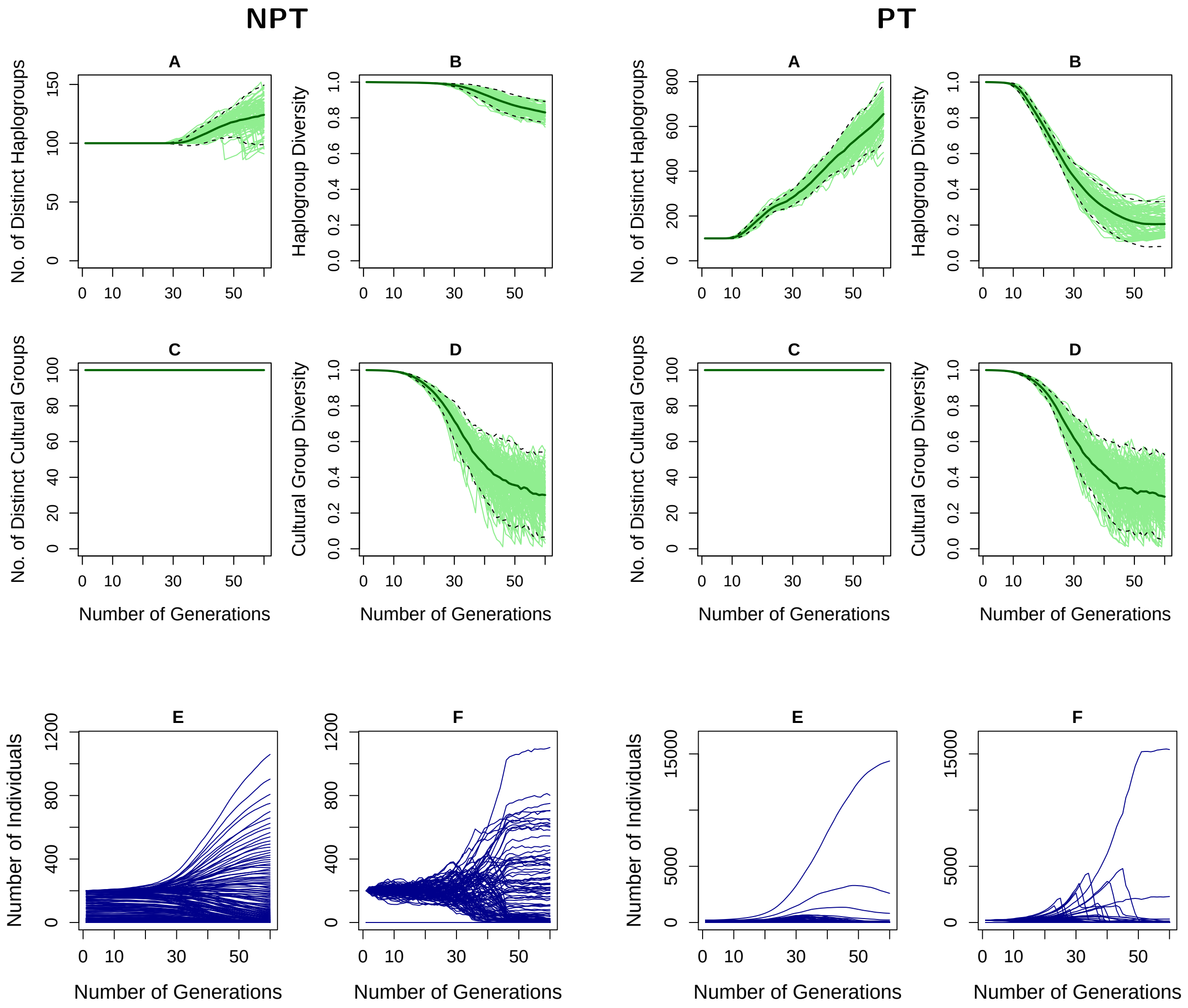


Supplementary Figure 20: Simulation with $N^{\text{total}} = 20000$, a choice of $\rho$ giving rise to a 25% average population decline rate for the first cultural group initially, and $|H| = 2500$. Left figures show results for initial complete non-patrilineality (NPT) while right figures show results for initial complete patrilineality (PT). (A)-(F) plot the same functions of the dynamics as plotted in Supplementary Figs 2-19.

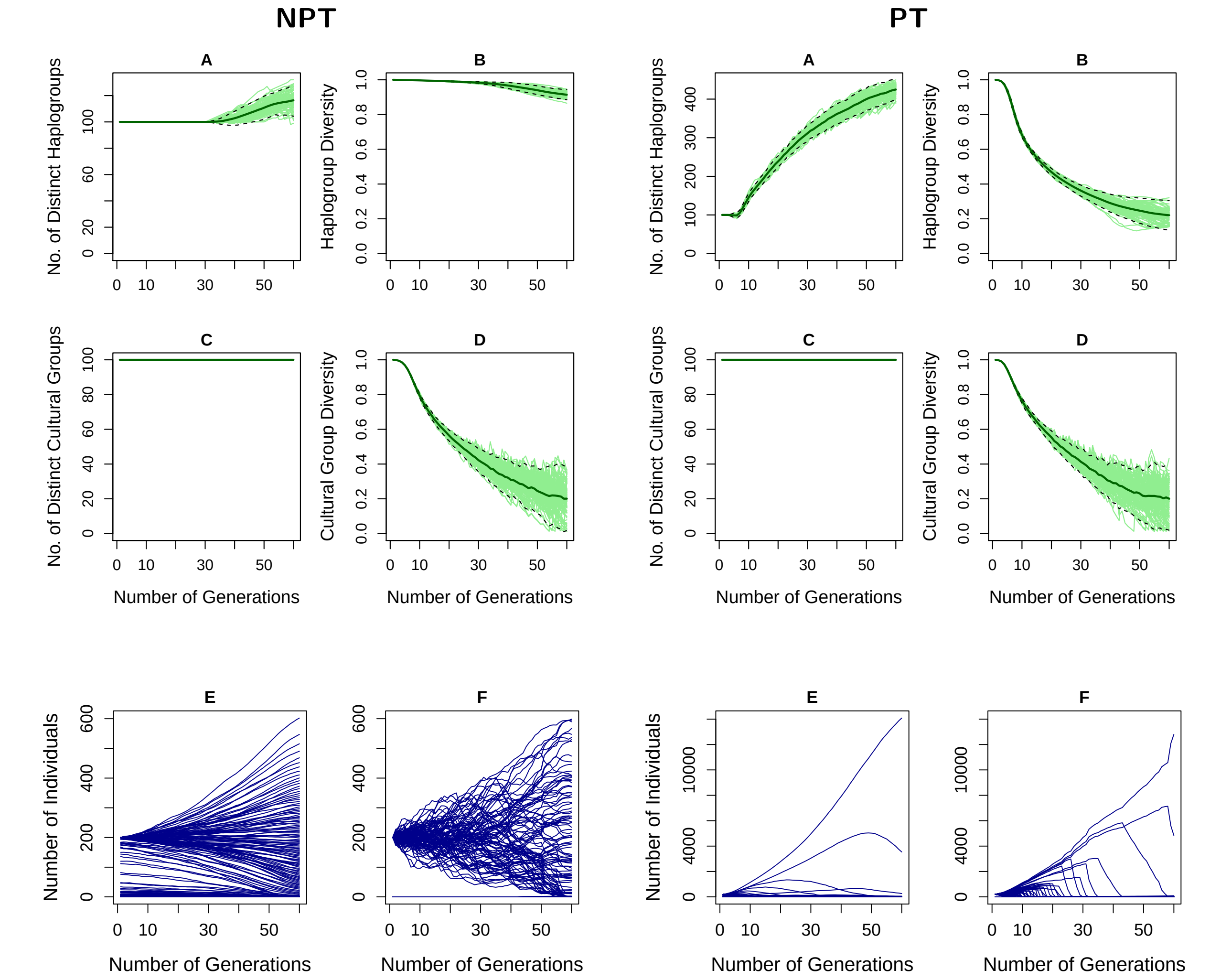


Supplementary Figure 21: Simulation with $N^{\text{total}} = 20000$, a choice of $\rho$ giving rise to a 25% average population decline rate for the first cultural group initially, and with selection differentiating fitness levels among cultural groups ($\alpha_c = 1.00+(c-1)\times 0.01$). Left figures show results for initial complete non-patrilineality (NPT) while right figures show results for initial complete patrilineality (PT). (A)-(F) plot the same functions of the dynamics as plotted in Supplementary Figs 2-19.

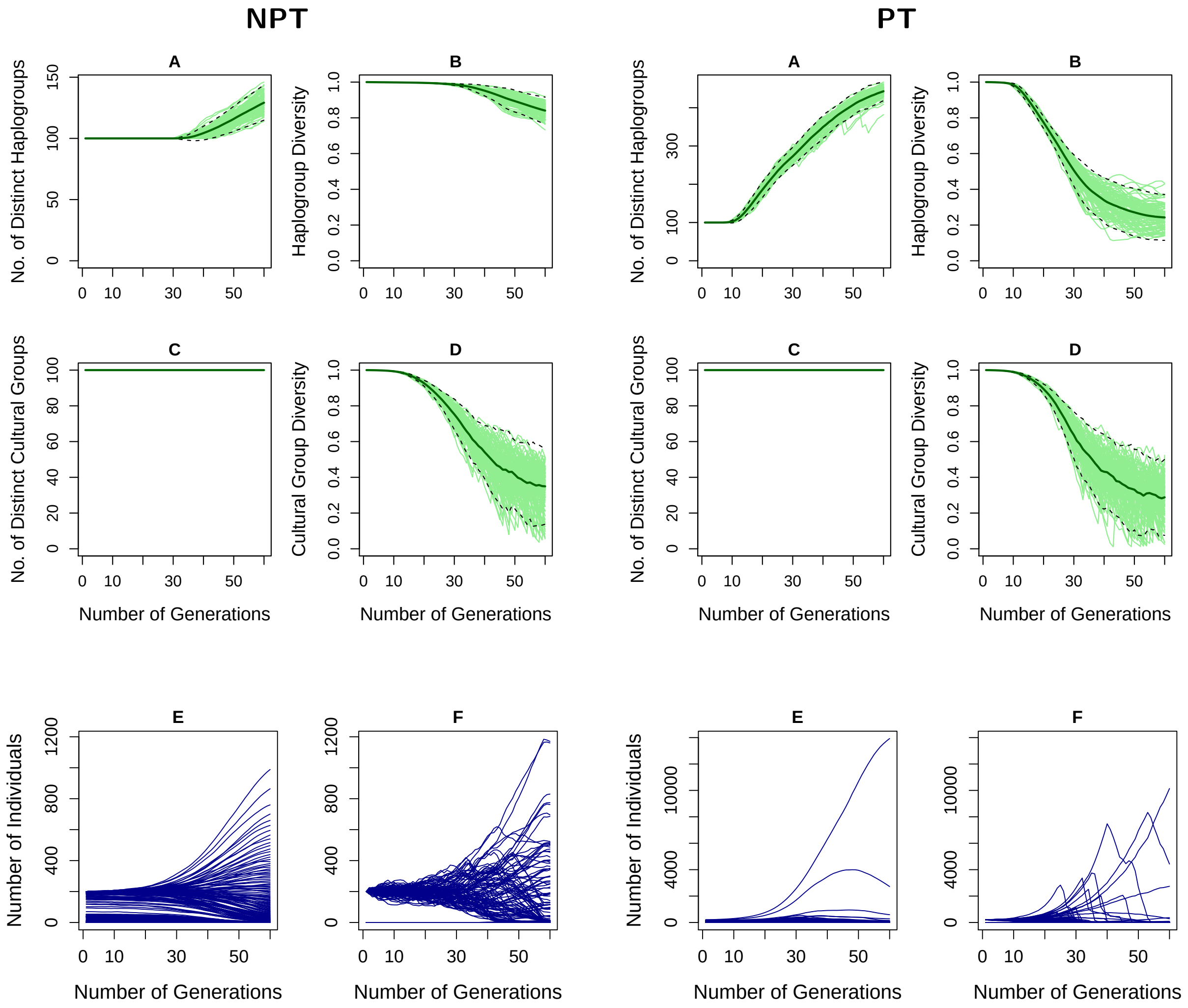


Supplementary Figure 22: Simulation with $N^{\text{total}} = 20000$, a choice of $\rho$ giving rise to a 25% average population decline rate for the first cultural group initially, with stepping-stone group fusion instead of weeding off small cultural groups. Left figures show results for initial complete non-patrilineality (NPT) while right figures show results for initial complete patrilineality (PT). (A)-(F) plot the same functions of the dynamics as plotted in Supplementary Figs 2-19.

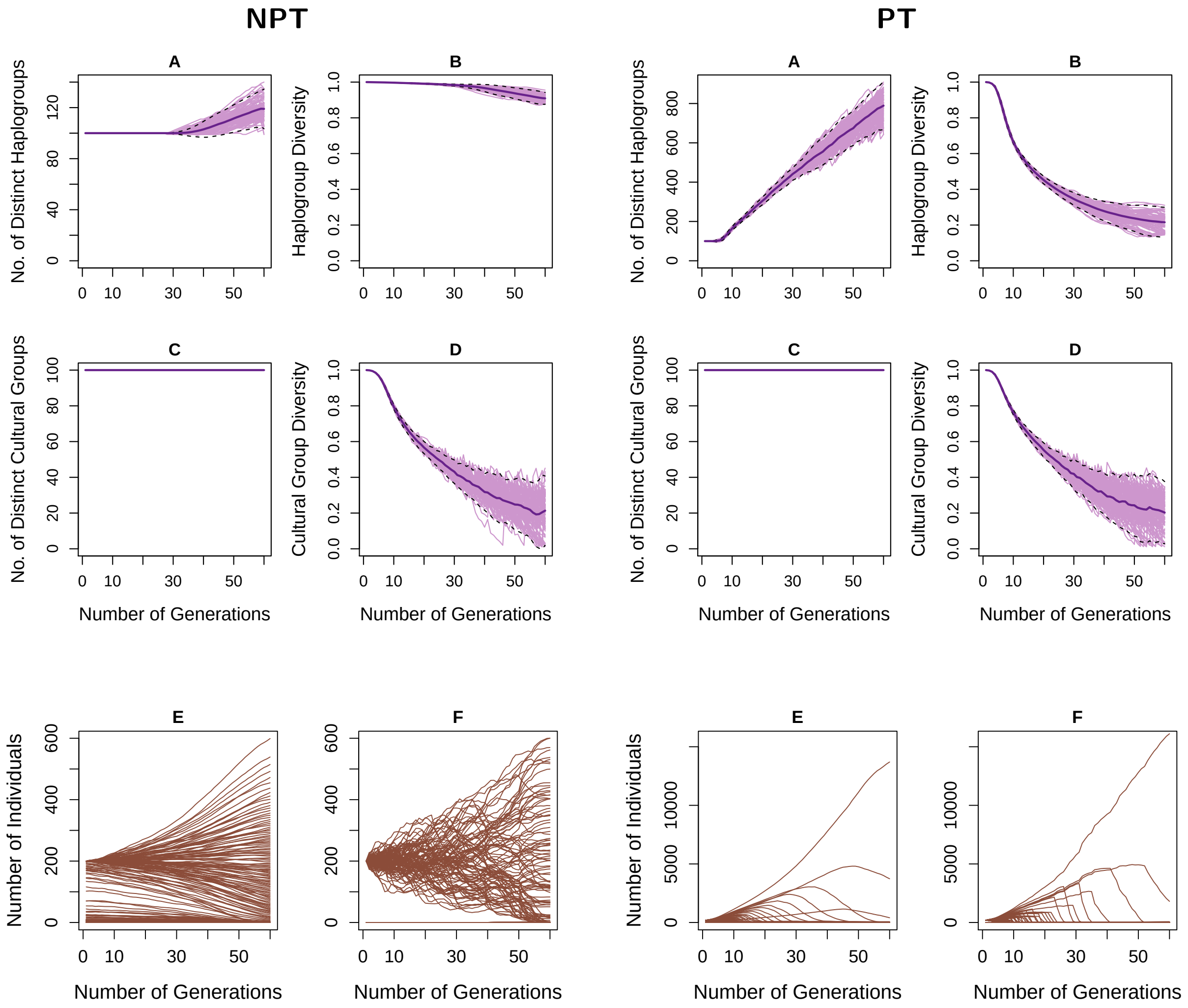


Supplementary Figure 23: Simulation with $N^{\text{total}} = 20000$, a choice of $\rho$ giving rise to a 25% average population decline rate for the first cultural group initially, with $|H| = 2500$, and with selection differentiating fitness levels among cultural groups ($\alpha_c = 1.00 + (c-1) \times 0.01$). Left figures show results for initial complete non-patrilineality (NPT) while right figures show results for initial complete patrilineality (PT). (A)-(F) plot the same functions of the dynamics as plotted in Supplementary Figs 2-19.

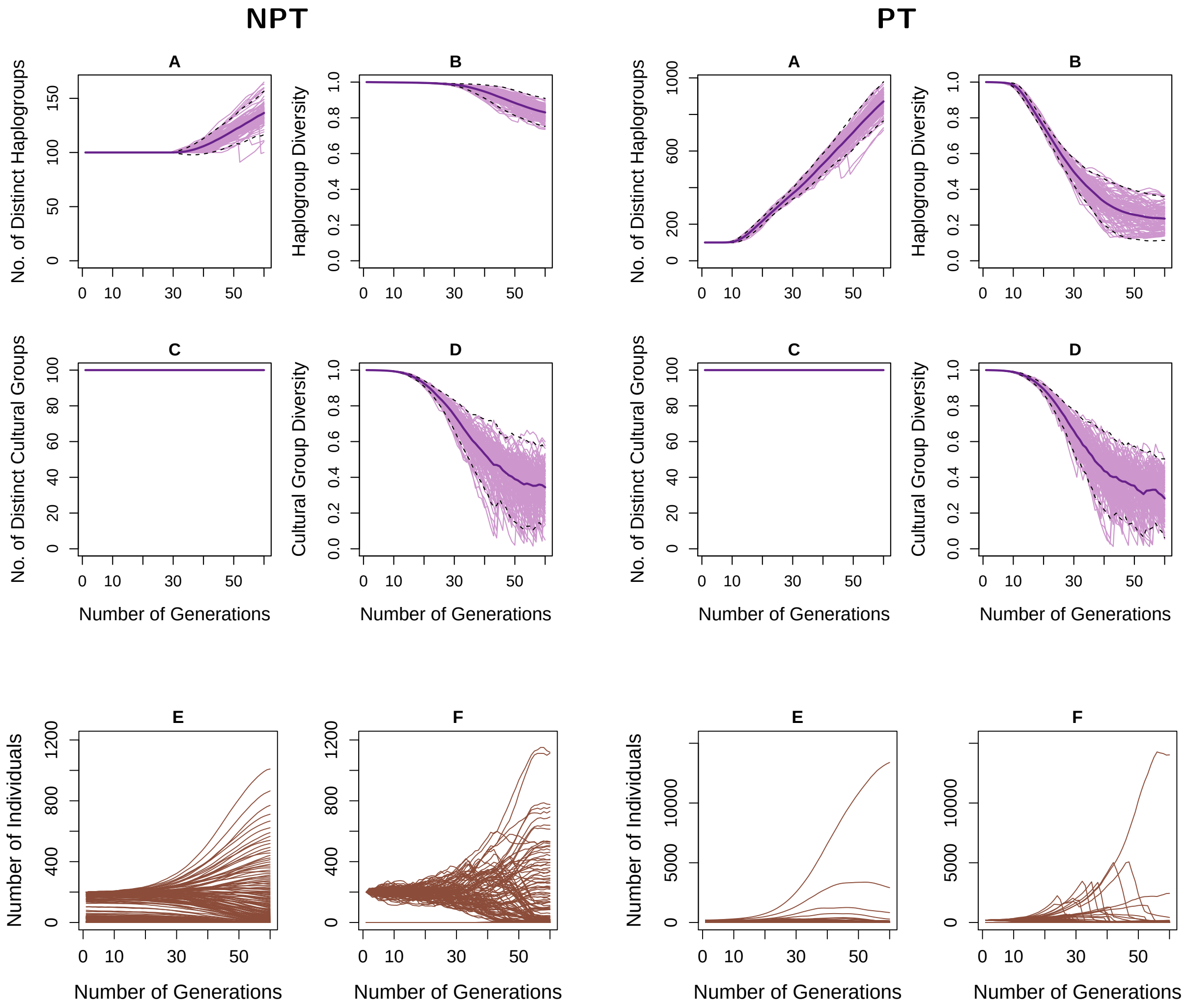


Supplementary Figure 24: Simulation with $N^{\text{total}} = 20000$, a choice of $\rho$ giving rise to a 25% average population decline rate for the first cultural group initially, with $|H| = 2500$, and with stepping-stone group fusion instead of weeding off small cultural groups. Left figures show results for initial complete non-patrilineality (NPT) while right figures show results for initial complete patrilineality (PT). (A)-(F) plot the same functions of the dynamics as plotted in Supplementary Figs 2-19.

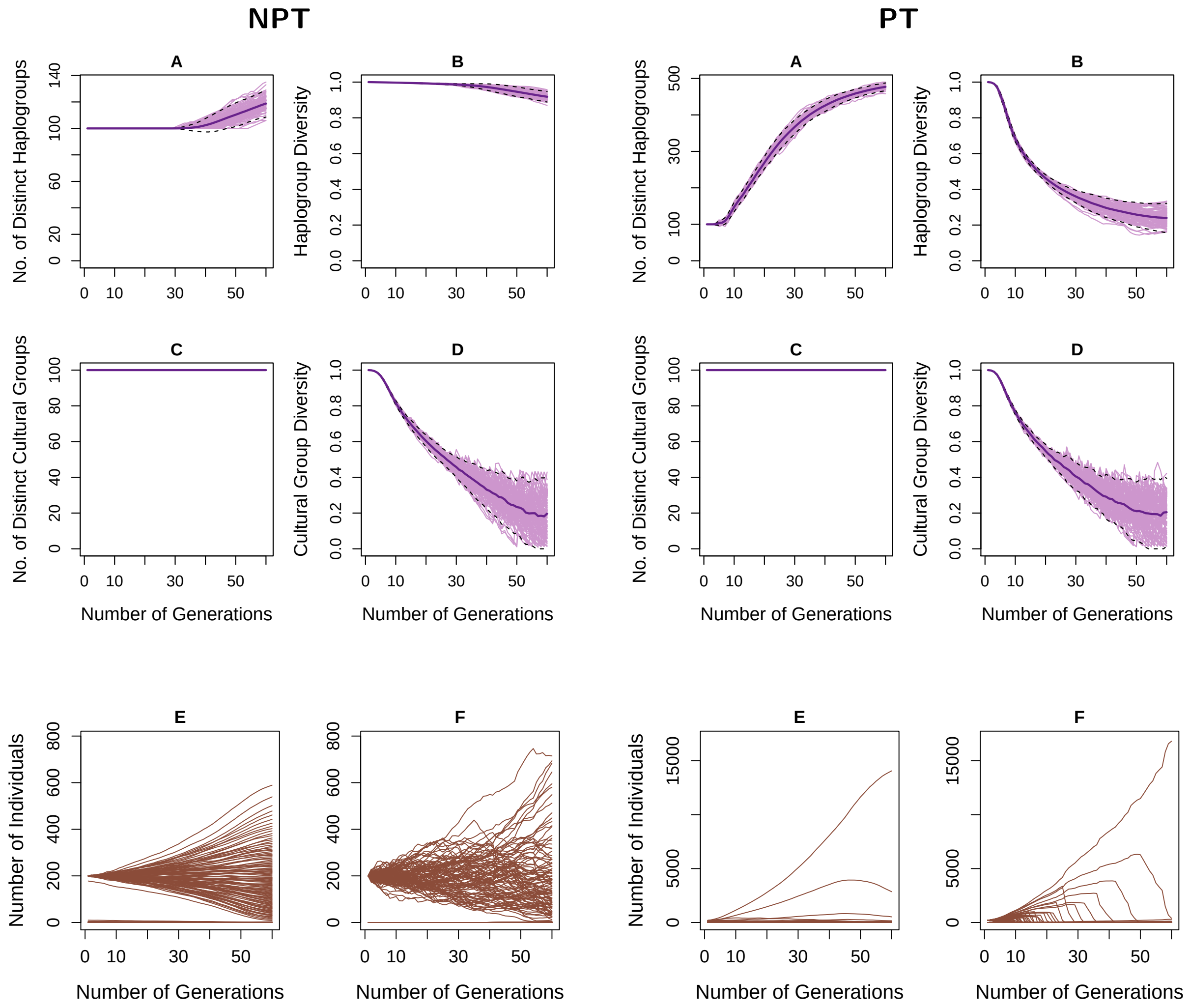


Supplementary Figure 25: Simulation with $N^{\text{total}} = 20000$, a choice of $\rho$ giving rise to a 25% average population decline rate for the first cultural group initially, with selection differentiating fitness levels among cultural groups ($\alpha_c = 1.00 + (c-1) \times 0.01$), and with stepping-stone group fusion instead of weeding off small cultural groups. Left figures show results for initial complete non-patrilineality (NPT) while right figures show results for initial complete patrilineality (PT). (A)-(F) plot the same functions of the dynamics as plotted in Supplementary Figs 2-19.

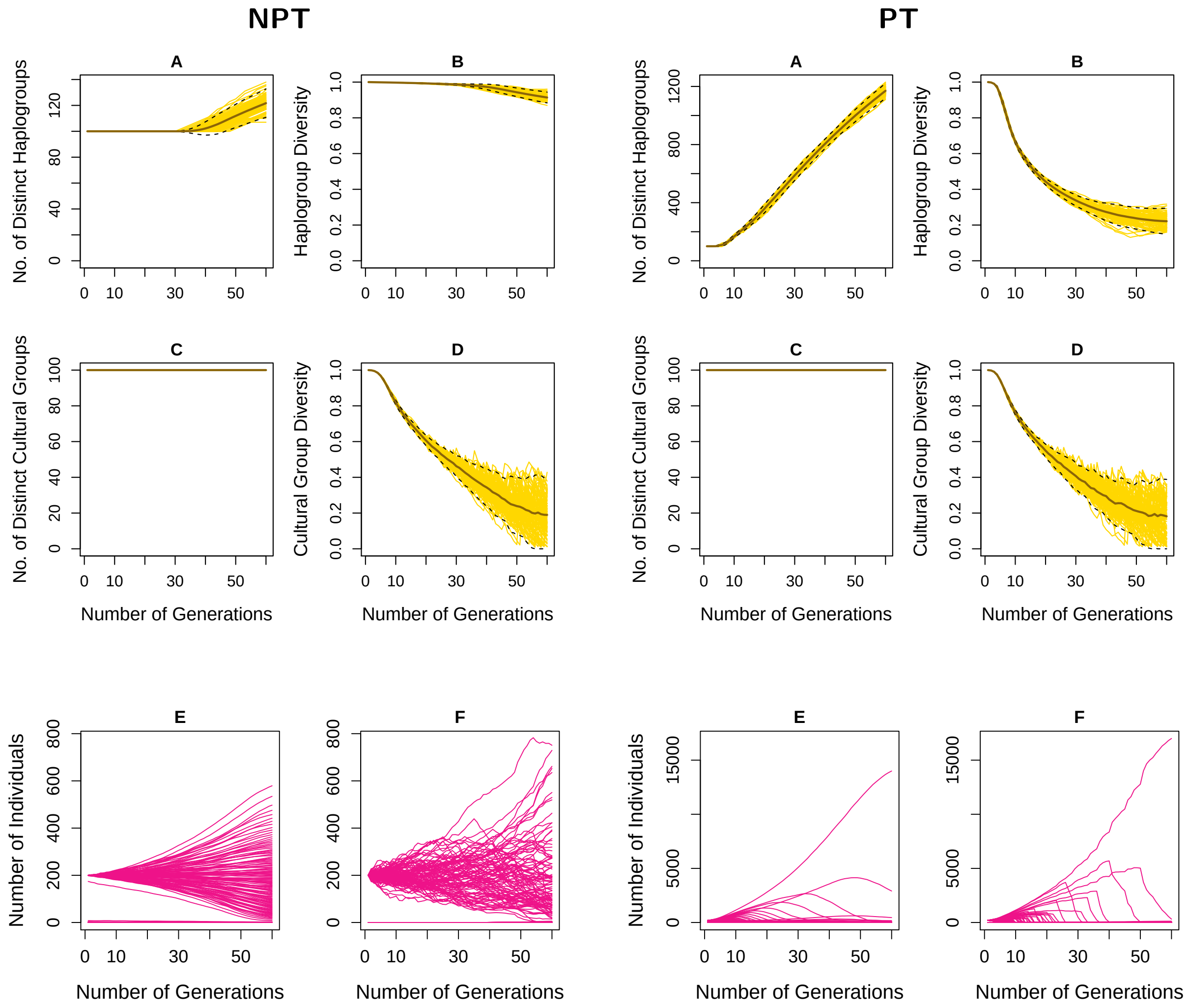


Supplementary Figure 26: Simulation with $N^{\text{total}} = 20000$, a choice of $\rho$ giving rise to a 25% average population decline rate for the first cultural group initially, with $|H| = 2500$, with selection differentiating fitness levels among cultural groups ($\alpha_c = 1.00 + (c-1) \times 0.01$), and with stepping-stone group fusion instead of weeding off small cultural groups. Left figures show results for initial complete non-patrilineality (NPT) while right figures show results for initial complete patrilineality (PT). (A)-(F) plot the same functions of the dynamics as plotted in Supplementary Figs 2-19.

## Supplementary Note 5: Stochastic Modifications to the Modified Lotka-Volterra Model

The modified Lotka-Volterra model (Supplementary Note 1) is arguably a poor representation of human population dynamics, since external factors not captured by the deterministic process may add a layer of uncertainty to the outcomes, even if the growth rate and competition parameters are known. We may modify eq. S1 to include effects of randomness by appending Brownian motions to each population in the system, so that the dynamics of each population are driven by an additional "random noise" proportional to the square root of its size. Mathematically,

$$\begin{aligned} dY_1(t) &= Y_1(t)\left(r_1X(t) - \sum_{i=1}^{n} c_{1i}Y_i(t)\right)dt + \sqrt{\gamma_1 Y_1(t)}dB_1(t) \\ dY_2(t) &= Y_2(t)\left(r_2X(t) - \sum_{i=1}^{n} c_{2i}Y_i(t)\right)dt + \sqrt{\gamma_2 Y_2(t)}dB_2(t) \\ &\vdots \\ dY_n(t) &= Y_n(t)\left(r_nX(t) - \sum_{i=1}^{n} c_{ni}Y_i(t)\right)dt + \sqrt{\gamma_n Y_n(t)}dB_n(t) \\ dX(t) &= aX(t)\left(1 - \frac{X(t)}{K}\right)dt + \sqrt{\nu X(t)}dB_{n+1}(t), \end{aligned} \tag{S4}$$

where $\{\gamma_i\}_{1\leqslant i\leqslant n}$ and $\nu$ are positive constants and $\{B_i(t) : t \geqslant 0\}_{1\leqslant i\leqslant n}$ is a collection of mutually independent standard Wiener processes.

We refer to eq. S4 as the **m**odified **s**tochastic **L**otka-**V**olterra **c**ompetition (MSLVC) process; it extends the stochastic Lotka-Volterra competition process, a model in which there is no female population $X(t)$ and the rates $r_iX(t)$ are replaced by positive growth rates $r_i$. The stochastic Lotka-Volterra competition process itself arises from a family of stochastic differential equations known as generalized Feller diffusion processes, which have received attention in population biology studies in recent years (Bansaye and Méléard, 2015; Cattiaux et al., 2009; Cattiaux and Méléard, 2010).

By mirroring the stochastic dominance argument (Theorem 1.1, Chapter 6 of Ikeda and Watanabe, 1989) in the analysis of the two-dimensional SLVC, it can be shown that each of the $Y_i$ in eq. S4 is dominated by a Feller diffusion with logistic growth. Hence, the MSLVC $(Y_1, \ldots, Y_n, X)$ is dominated by the process $(\widetilde{Y}_1, \ldots, \widetilde{Y}_n, X)$, where each $\widetilde{Y}_i$ is a Feller diffusion with logistic growth in which the logistic growth rate is driven by $X$. By applying a result by Lambert et al. (2005) about the almost sure (a.s.) convergence to 0 in finite time of Feller diffusions with logistic growth, we see that the MSLVC must itself hit $(0, \ldots, 0) \in \mathbb{R}^{n+1}$ in finite time (a.s.).

To analyze eq. S4 meaningfully, one may wish to investigate its behavior conditioned on the event $\{X(t) > 0\}$. There are various ways this might be done, but one numerical approach is to compare the expected diversity of the male populations over time against numerical simulations of the female population dynamics (which do not depend on the males). Similar to the theoretical analysis of $n = 2$ male populations for the modified Lotka-Volterra model in Supplementary Note 1, we consider the process

$$\begin{aligned} dY_1(t) &= Y_1(t)\left(r_1X(t) - c_{11}Y_1(t) - c_{12}Y_2(t)\right)dt + \sqrt{\gamma_1 Y_1(t)}dB_1(t) \\ dY_2(t) &= Y_2(t)\left(r_2X(t) - c_{21}Y_1(t) - c_{22}Y_2(t)\right)dt + \sqrt{\gamma_2 Y_2(t)}dB_2(t) \\ dX(t) &= aX(t)\left(1 - \frac{X(t)}{K}\right)dt + \sqrt{\nu X(t)}dB_3(t), \end{aligned} \tag{S5}$$

which can be expressed in more streamlined notation. Let $Z(t) = (Y_1(t), Y_2(t), X(t))^\top$. Then, eq. S5 can be written as

$$dZ(t) = \mu(Z(t))dt + \sigma(Z(t))dB(t), \tag{S6}$$

where $B(t) = (B_1(t), B_2(t), B_3(t))^\top$ is standard 3D Brownian motion satisfying

$$\sigma(z_1, z_2, z_3) = \begin{pmatrix} \sqrt{\gamma_1 z_1} & 0 & 0 \\ 0 & \sqrt{\gamma_2 z_2} & 0 \\ 0 & 0 & \sqrt{\nu z_3} \end{pmatrix},$$

and $\mu(z_1, z_2, z_3) = \left(r_1 z_1 z_3 - c_{11} z_1^2 - c_{12} z_1 z_2, r_2 z_2 z_3 - c_{21} z_1 z_2 - c_{22} z_2^2, a z_3 - \frac{a z_3^2}{K}\right)^\top$. If $f(z_1, z_2, z_3) = \frac{z_1}{z_1+z_2} \log\left(\frac{z_1+z_2}{z_1}\right) + \frac{z_2}{z_1+z_2} \log\left(\frac{z_1+z_2}{z_2}\right)$, then $f(Z(t))$ computes the (unnormalized) Shannon entropy index of the male population in eq. S6.

Applying the Itô calculus to compute $\mathbb{E}_{\boldsymbol{z}}\left[f(Z(t))\right]$, we must solve a partial differential equation (PDE) whose solution is equal to the expected value. This PDE is given in the following proposition.

**Proposition S1.** *Suppose that $u : \mathbb{R}_0^+ \times (\mathbb{R}_{>0}^+)^3 \to \mathbb{R}$ is bounded and solves the following PDE*

$$\begin{cases} \frac{\partial u}{\partial t} & = & \mathscr{L} u \\ u(0, \boldsymbol{x}) & = & f(\boldsymbol{x}) \qquad (\forall \boldsymbol{x} \in \mathbb{R}^3) \\ u & \geqslant & 0 \end{cases} \tag{S7}$$

*where*

$$\mathscr{L} = x_1(r_1 x_3 - c_{11} x_1 - c_{12} x_2)\frac{\partial}{\partial x_1} + x_2(r_2 x_3 - c_{21} x_1 - c_{22} x_2)\frac{\partial}{\partial x_2}$$
$$+ a x_3\left(1 - \frac{x_3}{K}\right) + \frac{1}{2}\left(\gamma_1 x_1 \frac{\partial^2}{\partial x_1^2} + \gamma_2 x_2 \frac{\partial^2}{\partial x_2^2} + \nu x_3 \frac{\partial^2}{\partial x_3^2}\right).$$

*Then, $u(t, \boldsymbol{z}) = \mathbb{E}_{\boldsymbol{z}}\left[f(Z(t))\right]$.*

*Proof.* We apply a martingale method to obtain the Kolmogorov backward equation.

Let $u^*(t, \boldsymbol{z}) = \mathbb{E}_{\boldsymbol{z}}\left[f(Z(t))\right]$. Because $0 \leqslant f(z_1, z_2, z_3) \leqslant \log 2$ for all $(z_1, z_2, z_3) \in (\mathbb{R}_0^+)^3$, $u^*(t, \boldsymbol{z}) \in [0, \log 2]$ for all pairs $(t, \boldsymbol{z})$. Let $\mathcal{F}_t = \sigma(X(s) : 0 \leqslant s \leqslant t)$. For all $s \in [0, t]$, the Markov property of Brownian motion implies that $\mathbb{E}_{\boldsymbol{z}}\left[f(Z(t)) \mid \mathcal{F}_s\right] = u^*(t - s, Z(s))$. This shows that $(u^*(t - s, Z(s)) : 0 \leqslant s \leqslant t)$ is a $\mathbb{P}_{\boldsymbol{z}}$-martingale[‡] with $u^*(0, \boldsymbol{z}) = f(\boldsymbol{z})$.

Suppose that $u$ is a solution of the PDE (eq. S7) that satisfies all the other analytical conditions in Proposition S1. Because $u$ is of class $C^{1,2}$ (i.e., differentiable w.r.t. $t$ and twice differentiable w.r.t. $\boldsymbol{z}$), an application of Itô's formula (Øksendal, 2003) produces

$$\begin{aligned} u(t-s, Z(s)) - u(t, \boldsymbol{z}) &= \int_0^t \left(\frac{-\partial u(t-s, Z(s))}{\partial t} + \mu(Z(s))^\top \cdot \nabla_{\boldsymbol{z}} u(t-s, Z(s)) \right. \\ &\qquad \left. + \frac{1}{2}\sum_{i,j=1}^3 b_{ij}(Z(s))\frac{\partial^2 u}{\partial z_i \partial z_j}(t-s, Z(s))\right) ds \\ &\qquad + \int_0^t \sum_{i=1}^3 \frac{\partial u(t-s, Z(s))}{\partial z_i} \sum_{j=1}^3 \sigma_{ij}(Z(s)) dB_j(s) \\ &= \int_0^t \sqrt{\gamma_1 Z_1(s)} \frac{\partial u(t-s, Z(s))}{\partial z_1} dB_1(s) + \int_0^t \sqrt{\gamma_2 Z_2(s)} \frac{\partial u(t-s, Z(s))}{\partial z_2} dB_2(s) \\ &\qquad + \int_0^t \sqrt{\nu Z_3(s)} \frac{\partial u(t-s, Z(s))}{\partial z_3} dB_3(s), \end{aligned}$$

[‡]That is, a martingale with respect to the $\mathbb{P}_{\boldsymbol{z}}$ measure; see Øksendal (2003) for details.

where $b_{ij}(\boldsymbol{x}) = [\sigma(\boldsymbol{x})\sigma(\boldsymbol{x})^\top]_{ij}$. Note that the second equality above follows from the identity $\frac{\partial u}{\partial t} = \mathscr{L}u$ in eq. S7 and from simplifying the expression in the stochastic integral. For the sum of the stochastic integrals in the last expression, which is a local martingale, to be a true mean-zero martingale, we must apply the boundedness condition. Because $u$ is bounded, the local martingale has finite quadratic variation; hence it is a mean-zero martingale. This implies that $\mathbb{E}_{\boldsymbol{z}}[u(t-s, Z(s))] = u(t, \boldsymbol{z})$ for all $s \in [0, t]$. Setting $s = t$, we obtain $u^*(t, \boldsymbol{z}) = \mathbb{E}_{\boldsymbol{z}}[f(Z(t))] = u(t, \boldsymbol{z})$. □

Using the PDE given in Proposition S1, we can compare the dynamics of male diversity up to simulated times at which the female population $X$ hits 0. Unfortunately, this approach is hindered by issues of convergence of numerical simulations for the female population dynamics $\{X(t) : t \geqslant 0\}$. Standard methods such as Euler-Maruyama lead to numerical solutions that diverge from the true solution for stochastic differential equations that fail to satisfy conditions on their drift and volatility coefficients. The Feller diffusion with logistic growth is one such equation (Hutzenthaler et al., 2011). In general, while stochastic differential equations provide a rich collection of biological models that capture effects of randomness not present in deterministic dynamical systems and also enable statistical inference (Fuchs, 2013), they can be mathematically and computationally intractable. A potential theoretical project would investigate ways of extending the modified Lotka-Volterra model (eq. S1) to incorporate effects of randomness, for instance via the stochastic differential equation route described above, and to show that incorporating randomness does not change the conclusion about the long-term behavior of the process.

## Supplementary Note 6: Other Computational Models

The Computational Grid Model (Supplementary Fig. 1), despite including both cultural group and haplogroup dynamics, is still limited in several aspects. The constant population size ($N^{\text{total}}$) assumption is unrealistic, and moreover the female population dynamics are not considered. We also do not consider deaths not arising from intergroup competition (e.g., epidemics or natural disasters), which would allow direct comparison of different scenarios within a single framework that could produce the evolutionary signal of the bottleneck and star-like expansion of lineages inferred from coalescent models. Below, we discuss two approaches that potentially generalize our treatment.

| | 1 ↓ | 2 ↓ | ... | S ↓ | C ↓ |
|---|---|---|---|---|---|
| $P_1$ → | 0 | 1 | ... | 1 | $A$ |
| $P_2$ → | 1 | 1 | ... | 0 | $A$ |
| ⋮ | ⋮ | ⋮ | ⋱ | ⋮ | ⋮ |
| $P_{n_1}$ → | 0 | 0 | ... | 1 | $A$ |

Cultural Group $A$

| | 1 ↓ | 2 ↓ | ... | S ↓ | C ↓ |
|---|---|---|---|---|---|
| $P_{n_1+1}$ → | 1 | 0 | ... | 1 | $B$ |
| $P_{n_1+2}$ → | 0 | 1 | ... | 0 | $B$ |
| ⋮ | ⋮ | ⋮ | ⋱ | ⋮ | ⋮ |
| $P_{n_1+n_2}$ → | 0 | 0 | ... | 1 | $B$ |

Cultural Group $B$

Supplementary Figure 27: A schema for modeling evolving strings. There are $|\mathcal{P}| = n_1 + n_2$ strings representing individuals who are partitioned into two cultural groups of size $n_1$ and size $n_2$. (The cultural group membership feature is encoded in the last column labeled C.) In the genetic component of these strings, there are S polymorphic sites and an outgroup individual is assumed to exist so that each position within an individual string is either '1' (derived allele) or '0' (ancestral / same as outgroup allele). The patterns of strings evolve over time (not shown above) and their evolution is determined by rules of competition, genetic mutation, and cultural group migration.

1. First Approach: Model Evolving Strings.

   Because the population bottleneck and subsequent star-like expansion of lineages inferred from coalescent models describe features of a population of lineages evolving under a Wright-Fisher framework, one possible way of verifying the hypothesis we proposed in the main text is to consider a huge population of sequences $\mathcal{P} = \{\mathtt{P}_i\}_{i=1}^{|\mathcal{P}|}$ partitioned into many different cultural groups, and to model the effects of intergroup competition and movement between cultural groups on $\mathcal{P}$ over time (see Supplementary Fig. 27). Each sequence $\mathtt{P}_i$ is a string of symbols representing one individual, where the symbols could encode either a nucleotide or a cultural feature (e.g., a symbol at a string position representing membership of a particular cultural group). This model can in principle track the effects of intergroup competition on molecular genetic variation — much like standard models of molecular evolution (Nei and Kumar, 2000) that track effects of mutation and recombination on molecular genetic variation in populations. To proceed, one may describe rules according to which sequences are removed from $\mathcal{P}$ (e.g., individuals who possess a sequence encoding a particular cultural or genetic motif are killed), or undergo genetic mutations, or move from one cultural group to another. At the end of the dynamical process, a (discrete-time) phylogenetic tree can be constructed from the remaining sequences in $\mathcal{P}$ using sub-sequence data corresponding to the genetic component. Haplogroups can also be retroactively defined based on shared sequence motifs, resolving the issue of defining "haplogroup mutation rate" in our current model. This tree can be compared to the phylogenetic tree obtained by Poznik et al. (2016). Such an approach is pursued by Fogarty et al. (2017) in studying of the evolution of cultural complexity, where cultural traits (the cultural analogue of a gene, see Cavalli-Sforza and Feldman, 1981) are modeled as bits in a string.

2. Second Approach: Incorporate Population Growth and Deaths.

   It is also possible to modify our current grid model to include both population growth and deaths of individuals not based on competition. To include population growth, one may allow $N^{\text{total}}$ to depend on the generation number $t$, with rules on the dynamic behavior of $N^{\text{total}}(t)$ (for example, $N^{\text{total}}(t)$ could be piecewise constant, an exponential function of $t$, etc.). A less controlled option is to directly model the reproduction of males, say, by assuming that at each generation an individual from cultural group-haplogroup $(c, h)$ produces a certain number of offspring. To include deaths not based on competition, one may consider imposing an additional tuning parameter that scales down the size of each cultural group-haplogroup $(c, h)$ after the killing and mutation steps in the model.

In either of the approaches suggested above, explicitly including the female population and modeling the interaction between the male and female populations are possible next steps. Apart from these two approaches, there may be other approaches, including agent-based models (Wilensky and Rand, 2015) and compartmental models (Godfrey, 1983) both of which are used widely in the social sciences and biology.

## Supplementary References


Oleg Balanovsky. Toward a consensus on SNP and STR mutation rates on the human Y-chromosome. *Human Genetics*, 136(5):575–590, 2017.

Vincent Bansaye and Sylvie Méléard. *Stochastic models for structured populations.* Springer, 2015.

Juan A Barceló and Florencia Del Castillo. *Simulating prehistoric and ancient worlds.* Springer, 2016.

Patrick Cattiaux and Sylvie Méléard. Competitive or weak cooperative stochastic Lotka–Volterra systems conditioned on non-extinction. *Journal of Mathematical Biology*, 60(6):797–829, 2010.

Patrick Cattiaux, Pierre Collet, Amaury Lambert, Servet Martínez, Sylvie Méléard, and Jaime San Martín. Quasi-stationary distributions and diffusion models in population dynamics. *The Annals of Probability*, pages 1926–1969, 2009.

Luigi Luca Cavalli-Sforza and Marcus W Feldman. *Cultural transmission and evolution: a quantitative approach.* Number 16 in Monographs in Population Biology. Princeton University Press, 1981.

Jack N Fenner. Cross-cultural estimation of the human generation interval for use in genetics-based population divergence studies. *American Journal of Physical Anthropology*, 128(2):415–423, 2005.

Laurel Fogarty, Joe Yuichiro Wakano, Marcus W Feldman, and Kenichi Aoki. The driving forces of cultural complexity. *Human Nature*, 28(1):39–52, 2017.

Christiane Fuchs. *Inference for diffusion processes: with applications in life sciences.* Springer Science & Business Media, 2013.

Keith Godfrey. *Compartmental models and their application.* Academic Press, 1983.

Agnar Helgason, Axel W Einarsson, Valdis B Gumundsdóttir, Ásgeir Sigursson, Ellen D Gunnarsdóttir, Anuradha Jagadeesan, S Sunna Ebenesersdóttir, Augustine Kong, and Kári Stefánsson. The Y-chromosome point mutation rate in humans. *Nature Genetics*, 47(5):453–457, 2015.

Joseph Henrich. Cultural group selection, coevolutionary processes and large-scale cooperation. *Journal of Economic Behavior & Organization*, 53(1):3–35, 2004.

Josef Hofbauer and Karl Sigmund. *Evolutionary games and population dynamics.* Cambridge University Press, 1998.

Martin Hutzenthaler, Arnulf Jentzen, and Peter E Kloeden. Strong and weak divergence in finite time of Euler's method for stochastic differential equations with non-globally Lipschitz continuous coefficients. *Proceedings of the Royal Society of London A: Mathematical, Physical and Engineering Sciences*, 467(2130):1563–1576, 2011.

Noboyuki Ikeda and Shinzo Watanabe. *Stochastic differential equations and diffusion processes.* Elsevier (North Holland Publishing Company), 1989.

Monika Karmin, Lauri Saag, Mário Vicente, Melissa A Wilson Sayres, Mari Järve, Ulvi Gerst Talas, Siiri Rootsi, Anne-Mai Ilumäe, Reedik Mägi, Mario Mitt, et al. A recent bottleneck of y chromosome diversity coincides with a global change in culture. *Genome Research*, 25(4): 459–466, 2015.

Turi E King, Stéphane J Ballereau, Kevin E Schürer, and Mark A Jobling. Genetic signatures of coancestry within surnames. *Current Biology*, 16(4):384–388, 2006.

Amaury Lambert et al. The branching process with logistic growth. *The Annals of Applied Probability*, 15(2):1506–1535, 2005.

Priya Moorjani, Sriram Sankararaman, Qiaomei Fu, Molly Przeworski, Nick Patterson, and David Reich. A genetic method for dating ancient genomes provides a direct estimate of human generation interval in the last 45,000 years. *Proceedings of the National Academy of Sciences*, 113 (20):5652–5657, 2016.

Masatoshi Nei and Sudhir Kumar. *Molecular Evolution and Phylogenetics*. Oxford university press, 2000.

Bernt Øksendal. *Stochastic differential equations*. Springer, 2003.

G David Poznik, Yali Xue, Fernando L Mendez, Thomas F Willems, Andrea Massaia, Melissa A Wilson Sayres, Qasim Ayub, Shane A McCarthy, Apurva Narechania, Seva Kashin, et al. Punctuated bursts in human male demography inferred from 1,244 worldwide y-chromosome sequences. *Nature genetics*, 48(6):593–599, 2016.

Marc Tremblay and Hélène Vézina. New estimates of intergenerational time intervals for the calculation of age and origins of mutations. *The American Journal of Human Genetics*, 66(2): 651–658, 2000.

Kenneth M Weiss and H Martin Wobst. Demographic models for anthropology. *Memoirs of the Society for American Archaeology*, 38(2):i–186, 1973.

Uri Wilensky and William Rand. *An introduction to agent-based modeling: modeling natural, social, and engineered complex systems with NetLogo*. MIT Press, 2015.
